# Clinical Review, and Hospital Reports

**Published:** 1836-10-01

**Authors:** 


					III.
Clinical Review.
Birmingham Infirmary.
]. Report of the Out-Cases at-
tended by George Parsons, Esq.
F.L.S., from January 1, 1834, to
Dec. 31, 1835.
2. Report of the Out-Patients at-
tended by Frederick Ryland,
Esq., between the 25th Decem-
ber, 1834, and the 25th Decem-
ber, 1835.
These Reports are creditable to the au-
thors, and evince a very praiseworthy
disposition on their part, to render the
facts that fall under their notice useful,
as the bases of statistical conclusions.
We feel much pleasure in thus publicly
complimenting Mr. Parsons and Mr.
Ryland, and in encouraging them to
persevere in this honorable labour.
They cannot benefit their readers, with-
out in a greater ratio benefiting them-
selves.
Let us first take up the Report of
Mr. Parsons. In the course of twelve
months, 2,975 cases came under his
observation. Of these, 141 proved fa-
tal. The deaths amongst the males
were 72?amongst the females, 69. Of
the males, 35 died under ten years of
age, and 37 above that. Of the females,
34 died under ten years of age, and 35
above that. The following table shews
the diseases and ages of the individuals
who died.
1836] Report of the Birmingham Infirmary. 529
Diseases and Ages of the 141 Fatal Cases.
Dentitio
Erythema marginatum..
Febris rem. Infantum ..
Pertussis
Variola
Convulsiones Infantum..
Marasmus
Scarlatina
Pneumonia
Laryngitis acutus
Diarrhoea
Synochus
Burn
Hydrocephalus acutus ..
Phthisis Pulmonum
Cynanche tonsillaris . ..
Fungus medullaris
Bronchitis acutus
Peritonitis acutus
Pleuritis
Mania
Epilepsia
Ascites
Bronchitis chronicus.. ..
Glottitis acutus
Anasarca
Scirrhous Pylorus
Purpura
Cholera biliosa
Incontinentia Urinas....
Hernia
Icterus
Apoplexia
Constipatio
Fractura
Males . .
Females.
Total ...
30
II
1 1
1 1
1 .. 1
1 3 4
3 3
4 2 6
2 9 11
12 11 23
4 .. 4
3 .. 3
1 6 7
10 3 13
1 .. I
1 2 3
13 10 23
1 1
1 1
1 .. 1
2
1 .. 1
1 .. 1
1 1 2
1 1 2
6 11
1 1
3 3
2 .. 2
1 .. 1
1 .. 1
1 .. 1
1
1 .. 1
1 .. 1
1
1
72 69 141
1 69
1 141
Now it appears that 51 of tliose who
. died were under five years of age, and
69 under ten years. None were engaged,
in any trade. Mr. Parsons, indeed, in-
forms us that, except in the pin-manu-
factories, it is not common for children
under ten years of age to be employed
in the workshops in Birmingham. Of
the 72 persons above the age of ten
years, only 43 were engaged in any em-
ployment, viz. 32 males, and 11 fe-
males.
It would be idle to found any con-
clusions on so small a number of cases,
and we therefore bmit the table which
shews the occupations of these 43 per-
530 Periscope j or, Circumspective Review. [Oct. 1
sons. Mr. Parsons, to render the list
more valuable, subjoins another table,
containing the trades of 134 persons,
who died in three years?in 1833,
1834, and 1835. Of the 134, 100 were
males, and 34 females. This, also, is
too small a number, and the deaths are
confined to too limited a period, to en-
able us to attach much importance to
such data. Yet they afford an intro-
duction to an acquaintance with the
diseases incidental to various trades in
Birmingham, and we accordingly sub-
join this table. The only diseases men-
tioned are consumption, chronic and
acute bronchitis, and continued fever,
for the deaths from other causes were
too few to require special notice.
:}
Tailors
Shoemakers
Clerks
Butchers
Labourers and Gardeners..
Masons and Bricklayers ..
Pedlars
Porters
Ostler
Barometer and Mirror
makers
Painter
Skinner
Carpenters and Cabinet-
makers
Brusli-maker
Whip-maker
Wood-turner
Tray-makers
Brass-founders
Brass-cock-founder ...
Button-maker
Pearl-button-maker ....
Lamp-makers
Gas-light-fitter
Tea-urn-maker
Tea-pot-maker
Platers
Jeweller
Chaser
Gilt-toy-maker
Smiths
Nailer
}
Wire-drawer
Whip-machine-makcr .
Steel-toy-makers
Spectacle-frame-maker
Bit-maker
Frying-pan-maker ...
Chain-maker
Buckle-maker
Gimlet-maker
Spoon-maker
Total
FEMALES.
Sempstress
Shoe-binder
Servants
Laundresses
Hucksters and Market-women
Coal-heaver
Brace-maker
Button and Plate Burnishers
Screw-makers
Stair-rod-maker
Japanner
Hook and Eye- maker
Lamp and Chandelier-makers
Plater
Nailer
Total
49 13
16 o 2! 3
It is not necessary to insert some
other tables shewing the periods of the
year and the ages at which bionchitis,
phthisis, and synochus, &c. respectively
appeared. Our acquaintance with the
laws of these diseases does not appear
to be much advanced by them.
In the years 1833, 1831, and 1835,
1836] Report of the Birmingham Infirmary. 531
there were 115 cases of small-pox, and
17 cases of it after vaccination?the
latter being to the former as 1 to 6.76.
In giving a table of cases of dyspep-
sia, we are informed that, out of 231
cases, 42 were in males, and 189 in
females. The disproportion between
the sexes in this respect, is certainly
not so great in the better classes. Has
gin-drinking any thing to do with the
indigestions of the Birmingham fair
ones ? A gin-shop proprietor in London
tells us that for one man who drinks
11111 cli of this fascinating liquid there
are at least two women. In Birming-
ham, the bowels of the ladies would
seem to be altogether out of sorts, for
We find in them 130 cases of diarrhrea
and six deaths, whilst the men present
only 74 cases and one death?amongst
the women were 13 cases of cholera,
and 12 amongst the men?in the former
47 cases of constipation, in the latter
^2?in the former 17 cases of hcemor-
rhoids, in the latter 11.
Dismissing these statistical records
Which appear to have been compiled
With equal care and industry, we shall
conclude by noticing a case or two de-
tailed by Mr. Parsons.
Case 1.?Medullary Sarcoma enter-
ing the Vertebral Canal.
A married woman, ret. 28, whilst
Walking in her house in the latter end
?f February, 1828, was suddenly seized
with complete loss of sensation and
voluntary motion, in all the parts of the
body below the middle of the chest.
The feces were ever after discharged
involuntarily, and it was necessary to
use the catheter daily. No disease of
the spine could be detected by the most
careful examination. A few days pre-
vious to her seizure, she had complained
of being slightly indisposed. We need
not mention the treatment, which was
perfectly inefficacious.
After the lapse of some weeks a
swelling became visible on the left side
of the spine between the scapula;, and
this tumour, which was tense and slightly
elastic under pressure, steadily, though
slowly, increased in size till it became
rather larger than a man's fist. It was
suspected to be a mass of the medullary
sarcoma, and it was not, therefore,
opened. Extensive sores formed on
the nates and back, but from them the
patient never experienced the slightest
uneasiness. She died on the 11th of
June, and the spinal columnwas opened
on the following day. The tumour was
found to be of the kind suspected. It
extended between the spinous processes
to the right side of the spine, and
passed into the spinal canal through
an aperture made by the partial des-
truction of the posterior portion of two
of the dorsal vertebrae, at which part
the medulla spinalis was disorganized,
being converted into a thin cream-like
substance.
The preceding is,pathologically speak-
ing, interesting. We have seen a case
very similar to it, but the paralysis was
gradually established.
The succeeding case of hernia is also
deserving of perusal.
Case IT.?Ventral Hernia?Strangu-
lation of the Bowel hy Omentum?Gan-
grcena Senilis in the Upper Extremity.
In the afternoon of the 15th of De-
cember, 1835, Mr. Parsons saw this
patient, a female, 63 years of age.
She complained of sickness and pain
in the bowels, and said that they had
not been opened for four days. . On
examination Mr. P. found a ventral
hernia immediately above the umbilicus,
and almost as large as a young infant's
head ; it was somewhat tender when
pressed, as was also the abdomen gene-
rally. Moderately firm pressure re-
duced the hernia to nearly half its size,
and a loud gurgling noise was heard
whilst the pressure was being made,
and likewise after it was discontinued,
when the tumour rapidly regained its
former magnitude. The hernia first
appeared about ten years since, and had
always been irreducible. From the pa-
tient's own account, it appeared that
she had on several occasions suffered
from similar attacks, which had always
been relieved by purgative medicine.
She had also suffered much from asth-
ma.
Mr. P. ordered calomel and cathartic
extract and clysters. She vomited the
former, and the latter seemed merely to
532 Phfiiscopk; or, Ciiicumspectivb Rkvikw. [Oct. 1
wash out the rectum; but she kept
some castor oil on the stomach.
In the evening there was more ten-
derness in the hernia and abdomen.
The pulse was very slow and feeble.
She now complained that she had lost
the use of the right arm, and that this
loss of power had come on, suddenly,
about an hour before the visit of Mr.
Parsons and his colleague, Mr. Bayn-
ham. The pulse was alike in both arms,
and the affected one was warm, and
the patient was able to move it freely.
The patient being in a state of much
prostration, and the intestine being
easily returned from the sac into the
abdomen, the operation was not per-
formed. During the night she vomited
frequently, the clysters returned only
slightly stained with feces, and next
day the hernia was larger. There was
now pulse in the right arm below the
upper part of the axillary artery, where
it was very feeble. The fingers and
hand were cold, shrunk, and livid, and
the surface of the whole of the fore-
arm was insensible, but she had much
pain in the elbow when it was bent.
An operation was now out of the ques-
tion. The hernia increased in size, the
mortification extended above the elbow,
and in the afternoon of the 18th she
died.
Dissection, twenty hours after death.
The brachial artery was very much con-
tracted, appearing to be not larger than
the radial ; it contained a small clot,
not adherent to its sides, which were
healthy. The coats of the subclavian
artery were also healthy, excepting im-
mediately below the clavicle, where
they were slightly diseased. There
was a dirty discolouration of the skin
over the hernia. In the sac was a large
mass of omentum, which was healthy,
but united to the sides of the sac by old
adhesions. Behind this omentum, and
also in the sac, was found a large fold of
small intestine, distended with air, quite
black and gangrenous. A finger could
be readily passed by the side of the
strangulated intestine, through the neck
of the sac, into the abdomen ; and the
gangrenous piece of intestine was, with-
out difficulty, pressed out of the sac
into the bellv. The intestine strangu-
lated, was a piece of the jejunum, anil
the cause of the strangulation was a
cord-like piece of omentum, about as
thick as a finger, which extended from
the abdomen into the hernial sac, where
it was adherent. Between this cord-
like piece, and a much larger mass of
omentum, likewise passing into, and
adherent to the sac, the fold of intes-
tine had slipped, and been compressed.
About six or seven inches of jejunum,
on each side of the strangulated por-
tion, were, like that, black and gan-
grenous, distended with flatus, and ap-
parently quite entire; but when cut
open with the scissors, the whole of
this part of intestine gave way like wet
paper. The intestines generally were
dark-coloured, and some folds of them
were adherent from the soft and recently
effused lymph, but this effusion was
neither large nor general.
An operation was not to be thought
of in this case, which we notice on ac-
count of the gangrene which occurred
in a distant part. This is not a very un-
frequent circumstance, especially in old
persons. It may take place however
in younger individuals of bad constitu-
tions, and especially in those addicted
to intemperance. We have seen one
lower extremity suddenly mortify after
amputation of the mamma, in a woman
between fifty and sixty years of age,
who was an habitual gin-drinker. We
have also seen mortification of one
lower extremity, after a scalp-wound,
in a man between thirty and forty years
of age, who had been in the habit of
drinking very large quantities of porter.
We might multiply cases but these are
sufficient to illustrate what we have
said.
We now quit the Report of Mr. Par-
sons, and turn to that of Mr. Ryland.
The cases to which it refers occurred
between the 25th of December, 1834,
and the 25th of December 1835. The
total number of out patients treated
during the year was 2173 ; the number
of deaths was 95.
Of the 2173 cases, 860 were males,
and the remaining 1313 females; of
the latter 576 were married, and 141
widows.
1836] Report of the Birmingham Infirmary. 533
. The deaths, during the year, were 95
,n nui*iber, 44 of which were males,
and 5] females. The relative mortality
? l-V? *"w.? sexes, at different ages, is
x 11"1ted in the following table :?
tfl
3 S.
Oi to
Under
1 year.
Between
1 and 5.
Between
5 and 10.
Between
10and20.
Between
20and30.
Between
30and40.
Between
40and50.
Between
50 and 60.
Between
60and70.
Between
70and80.
Total.
The deaths during the first ten yeais,
^m?unt to 45, or nearly one-half of the
?tal number of deaths ; of these 45, 22
^vere caused by scailatina and the sub-
sequent anasarca, 4 by bronchitis, 3
b>r small-pox, 2 by hydrocephalus, 3 by
fynochus, 2 by diarrhoea, and the rest
by more chronic diseases.
The succeeding table exhibits the age
and sex of 858 persons, affected with
eight diseases of the most frequent
occurrence.
No. L.
KOfJOCOOW
"< sr cr o cr 3
OOcO^wO^
l-i ? S3 5J O h?< jr* ?-*
C ? g. R E2. P 0 S--
" *T 5,(8 p ?? =" 5'
, ft ~ P 01
: c 2 a ; ? o ;
? 3 ? S: . g:
: ??: s-. : s:
I W I |
Mill-
OT to CO M w w w to
O* I 00 M 01 W
CO I to I o to
C^tOVCW^COHW
COHCOHtOtOWh-
I w I
to 00 H- o to ^ 00
it* tO I ?? CO to
to O) to I Ol to O Jl
s
& ?tj
?^5
n> a.
S =5
S8
SB
? w
<= ra
j? <J
P 3
p. ra
u> 2
o P
"a
P ft
|3
f2* o
?*? 2
? 3
11
So far as the preceding table goes, it
shews the greater prevalence of sickness
amongst females than males. This is
consistent with what we see in ordinary-
practice. But although there are more
women sick, more men die, at least the
statistical tables tell us so. The present
Report, however, shews an excess of
deaths on the female side. The follow-
ing analysis of the diseases affecting
persons who follow particular trades is
worth looking at.
Of 38 tailors, shoe-makers, and semp-
N N
534 Periscope ; ok, Circumspective Review. [Oct. 1
stresses, 23 were affected with dyspepsia,
9 with rheumatism, and 6 with chronic
bronchitis.
Of 31 persons engaged in making
hooks and eyes, carding buttons, stick-
ing pins, and in other sedentary occu-
pations, 20 were affected with dyspepsia,
5 with rheumatism, and 6 with chronic
bronchitis.
Of 38 domestic servants, chairwomen,
and washerwomen, all of whom were
females, 23 were suffering from dyspep-
sia and chronic gastritis, 11 from rheu-
matism, and 4 from chronic bronchitis.
Of 36 labourers and blacksmiths,
8 were affected with dyspepsia, 18 with
rheumatism, and 10 with chronic bron-
chitis.
Of 32 makers of metal-buttons, gilt
and steel toys, nails and screws, 14
were affected with dyspepsia, 9 with
rheumatism, and 9 with chronic bron-
chitis.
Of 30 gun-finishers and brass-foun-
ders, who generally work in covered
shops, 14 were suffering from dyspepsia,
3 from rheumatism, and 13 from chro-
nic bronchitis. Of 19 iron-founders,
who, by the nature of their occupation,
are much exposed to variations of tem-
perature, 5 were affected with dyspep-
sia, 8 with rheumatism, and 6 with
chronic bronchitis.
Of 16 japanners and lackerers, all of
whom were females, 10 suffered from
dyspepsia, 2 from rheumatism, and 4
from chronic bronchitis.
Of 15 pearl-button-makers, 7 were
affected with dyspepsia, 4 with rheu-
matism, and 4 with chronic bronchitis.
Although it is impossible accurately
to arrange the preceding facts in a tabu-
lar form, we may, with advantage, divide
the subjects of the three diseases into
two classes, viz. into those who, by
their occupations, are much exposed
to changes of temperature, and into
those who are sheltered from atmos-
pheric variations, by working in covered
rooms.
First class of cases..
Second ditto
Dyspepsia.
26
106
Rheumatism.
33
39
Chronic
Bronchitis.
Total.
18 77
44 189
The sedentary, as we might expect,
suffer most from dyspepsia, and the
females, the most sedentary, suffer from
it most.
Scarlatina would seem to have pre-
vailed much in Birmingham. It only
reached the district where Mr. R. at-
tends in August. It was at its height
in October and November, and declined
in December. The greatest number of
cases, 25, occurred in October, and the
deaths were in that month 5. All the
fatal cases occurred in children under
10 years of age.
St. Thomas's Hospital.
St. Thomas's Hospital Reports. By
John F. South, Assistant Surgeon.
These Reports are still published with
regularity, if not with success, and the
fourth number is now before us. This
same number completes a volume, the
first, and, we hope, not the lastthat shall
issue from within the walls of St.
Thomas's.
In the notice which constitutes the
preface to the whole work, we find the
following passage.
" A detailed account of cases occur-
ring in a hospital, to which several
physicians and surgeons are attached,
must be considered valuable, as afford-
ing a comparison of different modes of
practice, and their results. It is, there-
fore, matter of surprise, that no such
cases have hitherto been published by
any of the large hospitals with which
medical schools are connected, but have
been left to the periodicals, and by thei?
selected more with reference to rarity
than general utility, to the neglect of a
legitimate and ample field for profes-
sional improvement."
^836] Phlebitis. 535
General positions usually receive as-
sent, because they must be either ob-
viously true, or plausible, for their au-
thors to put them prominently forward.
There can be no doubt that the position
{aid down in the passage we have quoted
?s correct. A precise account of the
cases which occur in the great metro-
politan hospitals is highly desirable,
and must be useful. But it is not
enough that the cases are given, they
should be presented in a manner agree-
able to the public. It is very often on
the mode in which a thing is done, that
?ts success or failure hinges. Whether
the plan adopted in this instance is the
best that could be hit on, will be de-
cided by the event. For our own parts,
^e think that the cases are too detailed,
too directly transferred from the ward-
book to the press, and the observations
?f the medical officers too elementary
and common-place, for the profession
at large. We are not all students, at
'cast not young students, nor do we
Want to pay for being told what most
?f us are familiar with. Such hospital
reports as are calculated to go forth to
the medical world, should convey broad
generalizations, statistical documents,
new modes of practice, or novelties in
doctrine. We are far from approving
of the rage for extraordinary cases, for
horrible operations, for the monstrous
111 surgery and physic. Some of our
contemporaries would seem to have
'mbibed the dramatic taste of France,
?where a play must contain a score of
rapes, incests, unmentionable crimes,
and murders, to be palatable.
Uut although the appetite for bloody
?Perations and for rare cases is a vici-
ous one, it does not therefore follow
that all common cases are fit to be pub-
hshed. The profession must be allowed
to know something, and however anxi-
ous an editor may be to point out the
Propriety of studying ulcers of the leg,
bis readers will probably evince a natu-
ral indifference on the occasion. In
short, much judgment is required in
selecting appropriate subjects for clini-
cal publication, and as the public are
to be pleased, the public will decide on
what it likes, or does not care for. To
that public, therefore, we leave its own
business, and proceed to our's. We are
to give a sketch of the contents of the
work before us.
Those contents are?part of a case
of phlebitis, and remarks on it by Dr.
Roots?two cases of stone in the blad-
der, with a clinique by Mr. Tyrrell?
a case of acute pericarditis, with re-
marks by Dr. Roots?a report on stran-
gulation and mortification of hernia by
Mr. Tyrrell?a case of chronic laryn-
gitis, with remarks by Dr. Roots?three
cases of medullary carcinoma, with ob-
servations by Mr.Travers?and, finally,
a case of enuresis, with remarks by
Dr. Roots.
The reports on pericarditis, on laryn-
gitis, and on enuresis, have been already
noticed in the present number. We shall
say no more aboutthem, but take in order
the remainder as we have set them down.
I. Phlebitis.
The case that formed the subject of
Dr. Roots's remarks need not be related
here. Our readers are aware, that on
many occasions we have directed par-
ticular attention to phlebitis, and we
shall therefoie only allude to such pas-
sages in Dr. Roots's observations as
bear upon disputed points, or them-
selves admit of dispute.
1. What is the time at which severe
inflammation of a vein supervenes on
venesection ? Dr. Roots answers that
question thus:?
" You are all of you, I presume,
aware that perhaps one of the most
formidable conditions to treat which
comes under our care, is that of acute
inflammation of a vein supervening or
following upon venesection. The ordi-
nary symptoms, in the first instance,
are those only which are common to
inflammation. There is a pyrexial con-
dition?a local inflammation, and that
local inflammation manifested by pain,
redness, heat, and swelling; and, gene-
rally speaking, when following venesec-
tion, you have perhaps, sooner or later,
some purulent discharge from the punc-
ture of the wounded vein. But speedily
after these symptoms there is often a
most tremendous constitutional distur-
bance, commencing at various periods
N n 2
536 Pkriscopbj or, Circumspective Rbview. [Oct. 1
after the first inflammatory action. Mr.
Arnott states that it may vary from
two to ten or twelve days. Generally
speaking, so far as my own observation
goes, it has come on in from thirty-six
to seventy-two hours. I have rarely
seen much ^inflammation of the vein
with much constitutional disturbance
before what is called the secondary
symptoms, and which are commonly of
a typhoid character, have commenced,
or above seventy-two hours after the
first manifestation of inflammation in
the part. Speedily the symptoms as-
sume the character of typhus. There
is a quick, irregular, and feeble pulse,
a hot and dry skin, a loaded, brown and
dry tongue, and that disturbance of
the functions generally which you find
in fever of a low character."
Our own experience agrees with that
of Dr. Roots. We have not seen violent
inflammation of a vein from venesection
occur at any thing like so long an in-
terval as twelve days. But after am-
putations, which may, in one sense, be
considered venisection, we have seen
acute phlebitis follow at a distance of a
week to ten days from the operation.
2. Dr. Roots thus expresses his opi-
nion on the vexata qusestio?the cause
of the secondary purulent deposits.
" It has been supposed that these
depositions of pus, and this peculiar
disturbance of the system caused by in-
flammation of a vein, has been the re-
sult simply of pus being secreted in the
veins. I do not myself believe that
this is necessarily the case, because I
have seen some cases of death from in-
flammation of veins in which there was
no deposition of pus to be found in any
tissue of the body, excepting perhaps
some within the vein itself, and con-
sequently the deposition of pus is not
necessarily the result of pus mingling
with the ordinary fluid of the body?
the blood. Aware of this fact, Mr.
Arnott seems to wish to infer that these
secondary constitutional symptoms arise
either from the admixture of pus with
the blood, or, when pus is not secreted
by the inner coat of the vein, from some
morbid secretion, the result of inflam-
matory action ; that morbid secretion
acting very much the same, in inducing
the same effects and the same symP'
toms as the inoculation of animal poi-
son. There can be no question that
the symptoms do very much resemble
those which take place after the inocula-
tion, or absorption of an animal poison.
At present I do not myself believe it is
necessarily the result of pus being se-
creted ; but I agree with Mr. Arnott,
that it is the result of some diseased,
some morbid secretion, consequent on
the inflamed condition of the vein ming-
ling with the blood. At present, how-
ever, we cannot but acknowledge that
we are considerably in the dark as to
whether that is a sufficient explanation
or not."
No doubt the question is one of ex-
treme difficulty, and few have occasioned
more discrepancies of opinion. The
only chance of ariiving at the truth, is
by taking a wide and comprehensive
view of secondary inflammations, and
by continuing to make very careful dis-
sections. It is certain that the con-
dition of the smaller veins has not been
sufficiently investigated. We would
refer our readers to our notice of a
pamphlet on phlebitis by Mr. Douglas*
for a fuller account of the present state
of the question than is given by Dr.
Roots.*
3. "As far as I have been able to
judge, from looking at what others have
said on this subject?I may, however,
perhaps have mistaken them?it ap-
pears to me as if some entertained the
idea that these depositions of pus, found
either in the liver, in the lungs, in the
pleural cavity, in the cellular tissue, or
in the joints of the body, may be the
result of pur^ deposition, without the
previous existence of local inflamma-
tory action. I can only state to you
the result of my own experience. *
have seen many instances where these
deposits have taken place, some where
the patient has recovered, and others
where the case has terminated fatally*
but never did I see a case where puS
was deposited in an organ distant from
the seat of the vein primarily inflamed*
* The notice of Mr. Douglas' pam-
phlet is contained in our last number.
^36] Stone in the Bladder. 537
without manifest symptoms of inflam-
mation in that part."
There can be no doubt that Dr.
Roots is in the right. But, although
mflammation precedes the deposition,
the amount of that inflammation varies.
In one case, we find acute inflammation
both of the lung and of the serous
Membrane in the vicinity of the depo-
site; in other cases there are scarcely
any symptoms, and inspection after
death shews small evidence of inflam-
matory action. We have seen depo-
sites in the liver, with scarcely any
vascular injection of the parenchyma
around. The deposits in the subcu-
taneous cellular tissue are very fre-
quently attended with very slight in-
flammation. Usually a very faint ery-
thematous blush is seen upon the skin,
and a puffy or cedematous swelling is
occasioned by effusion of a small quan-
tity of bloody serum in the cellular
membrane. This effusion is gradually
superseded by pus, and the actual in-
flammation is by no means propor-
tioned to its quantity and character.
We have seen the knee-joint filled with
pus, without pain, and with the slight-
est injection of the synovial membrane.
On the whole, we would say that the
cases of purulent deposites exhibit great
varieties with respect to the quantum
of inflammation. On the one hand, we
see both the symptoms and the conse-
quences of violent and extensive inflam-
mation of a serous membrane?on the
other, we see pus almost insensibly
formed, with few of the phenomena,
symptoms, or anatomical effects of in-
flammatory action.
4. The treatment of phlebitis, and
that of the deposites, though sometimes
similar, must frequently be different.
We must remember that phlebitis is
usually a primary?the deposites a se-
condary affection. The distinction is
marked both in principle and practice.
Phlebitis, however it may commence
with decided inflammatory symptoms,
is yet apt to rather suddenly lead to
typhoid. This is more especially the
case when phlebitis is traumatic, and
when it occurs in debauched or irri-
table persons. When it happens spon-
taneously, or from the influence of cold,
the symptoms arecommonlymoreacute,
and the disposition to typhus less.
Phlebitis, attended with inflamma-
tory symptoms, may be treated as other
inflammations are. The prudent sur-
geon will feel his way, because he
knows the tendency to the occurrence
of low symptoms. Calomel and opium
are admirable assistants to general and
local depletion, and to blisters. The
latter are of value in all forms of phle-
bitis.
That form which soon runs into ty-
phus, traumatic phlebitis in individuals
with impaired constitutions, must be
managed with less active remedies.
Sometimes we may stimulate, frequent-
ly we must support at the time when
we are applying leeches and blisters.
But do what we will, these are bad
cases, and the secondary inflammations
and deposites too commonly ensue.
What is to be the treatment of the latter ?
That is more difficult and more unfor-
tunate still. If decided inflammatory
symptoms are present, cautious anti-
phlogistic treatment must be had re-
course to. In all cases blisters are
applicable, and in some of substantial
service. But these are melancholy
cases, and the advent of the symptoms
may generally be considered the death-
warrant of the patient.
II. Stone in the Bladder.
This is a long case, and the remarks
on it are longer. We shall materi-
ally abridge the former, select only
some portions from the latter, and make
a few observations of our own, in or-
der to supply what appear to be omis-
sions in the enumeration of the bad
consequences of lithotomy.
Case. Richard Bax, set. 31, a far-
mer's servant, was admitted Nov. 25th,
1835, with symptoms of stone in the
bladder, which had existed for many
years. On his admission, he complained
of occasional pain in the glans penis
after micturition ; great irritability of
the bladder, and a frequent desire to
make water when the bladder contained
no urine. At night the pain was more
severe, and he had incontinence of urine.
538 Pkkiscopk ; ok, Circumspkctivk Ukview. [Get. 1
Exercise produced much pain, and gave
rise to distressing tenesmus. The urine
was perfectly natural, and contained no
mucus. He also complained of a fre-
quent cough; the sputum was mixed
with florid blood, but not to any very
great extent; he had some tightness
across the chest, and on applying the
stethoscope to the right side, the mu-
cous rattle could be heard, but not very
distinctly. These latter symptoms were
relieved by blistering the chest, and by
the internal use of digitalis,Avithasmall
quantity of diluted sulphuric acid.
The health being so far restored, the
operation was performed on the 11th
of December in the usual manner, with
the probe-pointed knife. The incision
was clear and distinct, and the haemor-
rhage no more than usual. The ex-
traction of the stone was attended with
difficulty, owing to its enormous size;
the forceps slipped repeatedly, and the
operation was necessarily protracted.
A lithic acid calculus, weighing four
ounces, two drachms and two scruples,
and measuring three inches and a half
in length, and two and a quarter in
breadth, was extracted, and the patient
removed to bed in an exhausted condi-
tion. An opiate was administered.
Nothing particular occurred until
the 12th, at noon, when a little tender-
ness was found in the left iliac region.
The urine passed at intervals through
the wound, with small portions of
" grit."* In the afternoon, there was
slight coldness at times in the lower
extremities. In the evening, the pulse
was as frequent as 120, and the ten-
derness in the left iliac region remained.
He was a little better next morning,
but was much excited in the day of the
13th, by the cries of a patient. In the
evening, he seemed much depressed.
Dec. 14, 3 a.m.?Complains of more
tenderness of the abdomen towards the
lower part; the integument in the pe-
rinseum, and in the inguinal and iliac
regions, on both sides, is covered with
an erysipelatous blush, much more so
on the left side; pulse 132, more irri-
table, possessing less volume and pow-
er. He was ordered ammonia, but
during the day he appeared to get
worse, and in the evening he was
thought to be sinking. The ammonia
and nutriment were perseveringly exhi-
bited throughout the night, and, at 5,
a.m. of the 15th, an evident improve-
ment took place. The erysipelatous
blush diminished, but the tenderness of
the abdomen had increased. Small
doses of calomel and opium?ammonia to
be taken only when he felt low. During
the day the tenderness of the abdomen
continued, but was more exclusively
felt on the left side.
On the lGth, the unfavourable symp-
toms had diminished still farther. The
gums were slightly affected by the mer-
cury. In the evening, he was allowed,
at his own request, some porter " with
the chill off," and a little ginger. On
the 17th, the erysipelatous blush had
quite disappeared. On the 18th, the
sloughy surface of the wound in the
perineum was separating. There was
diarrhoea, which required astringents.
On the 19th, the tenderness in the
abdomen had quite disappeared. On
the 26th, a portion of slough separated
from the wound, and on the 28th a
larger portion, when the surface of the
wound looked healthy.
After this, he became not so well.
The wound was more sloughy, and
some coagulum was discharged. He
suffered from cough and muco-purulent
expectoration, attended with a little
dulness, and not very perfect respira-
tion beneath the left clavicle. The
bladder was twice injected, and a little
" grit," with some coagulum, dis-
charged. On the 13th of January,
however, we find him again improving.
But even when the 27th of that month
had arrived, the urine passed princi-
pally or entirely through the wound.
Injections of the bladder from time to
time with warm water were practised,
and an attempt was made to retain a
catheter in the urethra and bladder.
* Grit is not the most exact term
that could be selected. The reporter
should have specified the nature of the
sabulous matter, and stated its chemi-
cal composition.
1836] Stone in the Bladder. 539
But it occasioned too great irritation,
and was abandoned. Pus now made
its appearance in the urine.
On the 23d of April, he was dis-
charged, having been gradually im-
proving both in health and strength ;
but there was yet an opening in the
perinseum, and he still passed some pus
with the urine.
The remarks of Mr. Tyrrell on the
preceding case, which are adapted for
notice here, apply to the following
points.
1. The Mode of Operation.
"The patient was placed upon the
table in the usual position, the ankles
being fastened to the wrists, and in or-
der to keep him in a more restricted
position, and to give him less opportu-
nity of moving, the neck-ligature, or
one passing from round the neck under
the hams, was also applied, keeping the
knees well towards the thorax. A staff
of large size was passed through the
urethra, and the stone was felt directly
the staff entered the bladder. The dif-
ficulty was to pa?s the sound in so as
to get it to the left side, and rather be-
neath the stone. I had some little trou-
ble in doing this : for the bulk of the
stone immediately stopped the extre-
mity of the instrument when it entered
the bladder; but it was necessary for
me to get it in this position that I might
enter the bladder freely with the proper
mstrument, and make a free section of
the prostate.
The mode in which I then proceeded
with the operation was this: the staff
being held by one of my colleagues, I
put into the perinaium, beginning the
incision about an inch and a quarter
beneath the arch of the pubes, close to
the median line or raphe ; I then cut
freely downwards and outwards to-
wards my right side and the patient's
left, between the tuberosity of the is-
chium and the anus, making the ex-
treme of the incision, I think, rather
below the posterior margin of the anus.
Having thus divided the integuments
and superficial fascia, and wounded a
branch of the superficial perinaeal arte-
ry (which did not, however, afford hae-
morrhage of consequence), I then pas-
sed my finger to the upper part of the
wound, felt the triangular fascia which
fills up the pubic arch, and gives pas-
sage to the urethra, perforated this near
the median line, and- thence was led to
the under part of the membranous por-
tion of the urethra. I extended this
incision by dividing the fascia freely in
the direction in which I had made the
external incision ; and in doing this, I
cut through the transversus perinsei
muscle, and of course the transverse
perinseal artery. I next passed my fin-
ger further into the wound, and, de-
pressing the rectum towards the sacrum,
I passed the knife to the membranous
part of the urethra, and having felt the
groove of the staff with the point of the
knife, I ran the knife along it as far as
I considered the situation of the pros-
tate, carrying my finger on and keeping
the rectum depressed to prevent injur-
ing it. I then changed the scalpel for
a long, slender beaked knife, adapted to
the groove of the staff; and having
passed the beak of the knife into the
groove, I took the staff into my own
hand, and guided by the groove, passed
the knife into the bladder, its edge be-
ing directed horizontally towards the
ischium, on the patient's left side, not
downwards in the direction of the ori-
ginal wound. The staff was then given
to my colleague again, and at the same
time I expressed a wish that he should
hold it well up, keeping it towards the
arch of the pubes as much as he could,
that it might be out of the way of the
further steps of the operation (the knife
at this time being in the bladder). I
then placed my finger in the lower part
of the wound upon the rectum, passed
it on till I felt the prostate gland, and
felt of course that portion of the knife
which was within the prostate. I then
cut freely in nearly in the direction of
the external wound so as to divide the left
lateral lobe of the prostate, and with
this I might have divided some portion
of the levator ani, or a few fibres might
have been divided as I passed the knife
in. I then carried my finger in to feel
the aperture, and found it to be as free
as I desired. The knife was then with-
drawn, and the staff also, because I
found I could easily reach the cavity of
540 Pbriscopb ; or, Circumspectivr Rkvikvv. [Oct. 1
the bladder with my finger, otherwise
I should have kept the latter as a guide
to the forceps. I passed in the forceps
on my finger, making it the director,
but the end of the forceps was obstruc-
ted in the bladder by the bulk of the
stone, and the bladder contracted upon
it, so that I was obliged gradually to
press the forceps into the bladder, which
I did above the stone, and then gradu-
ally extended the blades to overpower
the contraction of the muscular coat.
This I did before I attempted to seize
the stone, in order to give me space to
perform this part of the operation. I
then grasped the stone, but the open-
ing of the handles of the forceps show-
ed that the extreme blades were also
widely separated, so that I thought it
best to make an examination first, to
see whether I had got the smallest or
the largest diameter of the stone. I
found I had got a large diameter, I
therefore separated the blades, and with
the point of my finger soon succeeded
in turning the stone till I got it in the
smallest diameter, with the extremity
towards the opening in the perina:um.
The difficulty of extracting it resulted
from there being such a considerable
thickness of stone, that a small portion
of it only was held by the blades of the
forceps, and the grasp was not suffici-
ent to hold firmly. I had come well
provided, for I knew from previous ex-
amination that I had a large stone to
deal with. The forceps with which I
eventually succeeded are of a very in-
genious construction, more especially
as regards the hinge part of them, so
that instead of just opening the extre-
mities as in a common pair of scissors
or forceps, when the extremity is pres-
sed upon, as in seizing a stone, the
blades are thrown nearly parallel to
each other, or to such an extent as to
diminish very much the separation of
the external points. This construction
of the forceps enables the blades to lay
hold or come in contact with a much
larger portion of the stone itself; they
are also very rough on the inner surface
of the blades. I eventually succeeded
in extracting the stone with them. The
mode in which the forceps frequently
slipped was the best proof that I was
not using any extreme violence, for if I
had, from the position in which I stood,
I should, upon the forceps quitting the
stone, have gone back. But I had a
perfect command of myself, becausc
the force I was using was of moderate
extent, the object being to overcome
the resistance of the soft parts, rather
than forcibly to tear or separate them.
The stone was thus extracted."
Mr. Tyrrell is partial to this n*>de of
operation, because he has been very
successful with it. He has operated
forty-one times, and only lost one pa-
tient, which was in consequence of ery-
sipelas. This is a reason which is al-
ways satisfactory to the individual.
His own success is his best encourage-
ment, and he is naturally averse to
change a procedure, which has been to
him a fortunate one. Our own opinion
is, that the kind of instrument that he
employs signifies comparatively little
to the Iithotomist, provided he is expert,
and habituated to its use. We see ope-
rators boasting of success with instru-
ments of the most opposite construc-
tion?the knife?the gorget?and the
bistoire cachee.
It has long been a question, whether
it is safer to divide the prostate and
neck of the bladder freely, for the ex-
traction of a stone of some dimensions
?or to tear those parts in preference
to their division. There is much to be
said on both sides, and surgeons of
great experience are ranged on either.
Mr. Tyrrell is against laceration.
" It has been said, and perhaps with
some reason, that some ofMhe most
successful operators have not used a
gorget with a cutting extremity so large
as those generally in use, so that the
opening made in the bladder must have
been more limited, and in the extraction
of a large stone the wound must have
been principally of a lacerated charac-
ter, especially in the prostate. But I
should say, that according to the force
necessary in extracting a stone, so, ge-
nerally, in my own experience, has
been the severity of the after-symp-
toms.
2. Dangers of the Operation.
The dangers of the operation in
1836J Stone in the Bladder. 541
entering the bladder I consider to be
twofold. The first, in my own opinion,
consists in dividing the artery of the
bulb. The transverse artery is neces-
sarily divided; but if the incision be
commenced in the perinteum so high
up as to wound the bulb, or the com-
mencement of the corpus spongiosum,
the artery of the bulb is also wounded.
The operator sometimes gets too near
the ramus of the ischium in incising,
and if he do, he cuts the artery of the
bulb shortly after it passes from the
pudic artery. The direction in most
of the works upon lithotomy is, that
the operator is to cut between the ac-
celerator urina: of the bulb and the crus
penis, but he has no occasion to go so
high up : if he do, he is very likely to
Wound the artery of the bulb at so short
a distance from the trunk, that haemor-
rhage difficult to control may take place.
This, then, is the first danger, and it
may be avoided by commencing the in-
cision an inch and a quarter below the
pubes in the adult, and at a compara-
tive distance, according to the age of
the patient below that period of life.
The next danger is from wounding
the rectum. If the operator, in cutting
from the membranous part of the ure-
thra to divide the soft textures, cuts too
much down with the scalpel, the rectum
is pretty sure to be wounded. In di-
viding the soft parts, he can cut out-
Wards, and a little downwards, towards
the tuberosity of the ischium, and at
the same time keep the finger pressed
on the rectum to protect it.
These are the only dangers, I think,
from the operation?all others, I be-
lieve, are more imaginary than real.
With regard to wounding the internal
pudic artery, if you look at the instances
the dissecting-room, in the dead-
house, and in dried preparations, you
will hardly find a case in which it could
be cut fairly in lithotomy. It passes
behind the ramus of the ischium, pro-
tected by it, and could only be injured
hy the operator cutting round and draw-
ing the knife from within to without.
Independently of these circumstances,
injury to the artery of the bulb, and in-
jury to the rectum, I consider the ope-
ration of lithotomy, so far as regards
the opening of the bladder, so perfectly
simple, that there is hardly an opera-
tion performed on the human subject
that requires perhaps less skill when
the structures are well known."
Division of the artery of the bulb is,
no doubt, a bad thing, but no doubt,
also, it is very frequently committed,
without injurious consequences super-
vening. The directions with respect
to the spot in which the urethra is to
be cut into, are sufficiently precise?the
point in which the urethra is opened is
not quite so constant nor exact. Sur-
geons are told to cut into the staff's
groove in the membranous part of the
urethra. In a deep perinseum, this is
not always so easy ; at all events we
have found, on examining the parts
both after the operation on the living
body and the dead, the bulb, and of
course its artery, more or less divided.
We have seen this in more than one
instance, where no hemorrhage of any
importance had occurred. It is advis-
able, on all accounts, to avoid wound-
ing the bulb ; but, no doubt, it often is
wounded without material mischief.
Mr. Tyrrell adverts to another source
of haemorrhage?notorious, from the
death of a patient who was operated on
at the Middlesex Hospital, by a very
worthy gentleman and excellent ana-
tomist?the late Mr. John Shaw.
" There is sometimes a peculiarity
in the distribution of the arteries to the
penis, a circumstance noticed first by
Tiedemann, and delineated in his plates
of the arteries. The dorsal artery of
the penis, in that instance, instead of
passing as a branch of the internal pu-
dic, was derived from the internal iliac,
passed by the side of the bladder, by
the side of the prostate, by the side of
the membranous part of the urethra,
then beneath the arch of the pubes,
and onwards to form the dorsal vessel,
as when derived from the pudic artery.
It is perhaps curious that the first in-
stance of haemorrhage from a division
of this kind occurred to a gentleman
who, in a work he published on ana-
tomy, made some severe strictures on
losing patients from hEemorrhage in li-
thotomy. I am alluding to one who is
now no more, and perhaps ought to
542 Periscope; or, Circumspective Review. [Oct. 1
regard the maxim de mortuisnil nisi ho-
num; but I am not speaking in dispa-
ragement of this gentleman, but as a
matter of utility for your own caution.
After publishing strictures upon hae-
morrhage connected with lithotomy, the
first case that he operated upon, when
he became connected with a public hos-
pital, he lost from haemorrhage. Upon
dissection this variety was found, but
although he had published on the sub-
ject of the operation, he was ignorant
of the variety existing at all, notwith-
standing the plates of Tiedemann had
been presented to the world some years;
access, however, to them had not been
generally available in this country."
The following are Mr. Tyrrell's re-
commendations with respect to the
treatment of haemorrhage.
" The hemorrhage, in the ordinary
performance of the operation, is gene-
rally checked by keeping the patient a
little cool, and using moderate pressure.
If there should be hemorrhage from
such an artery as that of the bulb, I
think it would be best at an.early period
to have recourse to the application of
the cautery, if you found the applica-
tion of a ligature could not be made."
Mr. Tyrrell takes no notice of what
we believe to be a more frequent source
of bleeding than either of those already
mentioned. We allude to haemorrhage
from the vesical plexus of veins in old
persons. These veins become at times
exceedingly enlarged?lying on the
prostate, they must be necessarily more
or less divided?and, occasionally, they
give rise to a serious, and even to a fa-
tal haemorrhage. There are few sur-
geons in extensive practice, who have
not met with cases of this nature. We
heard the other day of an instance, in
which the crushing of a small calculus
in the bladder proved mortal, in conse-
quence of bleeding from enlarged veins
in the mucous membrane.
Wound of the rectum in lithotomy is
not so unfrequent as perhaps might be
supposed. We have seen the bowel cut
several times, but in every instance the
wound healed, without the occurrence
of recto-vesical, or recto-urethral fis-
tula.
Mr. Tyrrell does not rank among the
dangers of the operation, those acci-
dents that have at times been witnessed.
The gorget has transfixed both walls of
the bladder?the bladder has been
pushed before that instrument, and se-
vered from the urethra?the knife or
gorget has been carried between the
bas-fond of the bladder and the rectum.
Probably he deems these mere results
of ignorance, or awkwardness, or tre-
pidation ; and probably, also, he is
right.
3. Difficulties of the Operation.
" What, then, are the difficulties
which may be said to arise in the ope-
ration of lithotomy ? I should say that
the principal one in the present in-
stance was, from there being a large
stone, and the drawing of this through
the limited space afforded by the aper-
ture in perinseo. I had ascertained
pretty well the size of the stone before
the operation ; I gave it as my opinion
that it was about the size of a large
hen's egg, but it was perhaps altoge-
ther rather larger than this. I had no
doubt I could, without the use of ex-
treme force, get the stone through the
common aperture made in the lateral
operation. This would have been with
more ease accomplished had I been fur-
nished perhaps with instruments of a
different kind. The forceps with which
I at length succeeded had the peculiarity
I have already mentioned with regard
to the hinge, so that the blades are
thrown more parallel to each other
when the object is grasped by their ex-
tremities. A suggestion of .a useful
kind was made to me some few days
after the operation. Having an oppor-
tunity of meeting Mr. Liston, and speak-
ing to him about the operation, and
the difficulty I had from the slipping of
the forceps from the stone, he asked
me whether I had used forceps, the
blades of which were lined with linen.
The suggestion is an extremely good
one, as the hold would be much more
firm than with a metallic substance.
The difficulty then arose from the ex-
traction of the stone through the aper-
ture, and I should urge it strongly upon
you not to use any great violence, but
to keep up a moderate degree of pres-
1836] Stone in the Bladder. 543
sure when you get a firm hold of the
stone, and to allow the parts to become
relaxed rather than lacerate them by
the force of extraction.
Another difficulty met with in my
own practice has been from the stone
being extremely small. You observe
that the extremities of the forceps do
not quite touch, and this is for the pur-
pose of avoiding nipping the mucous
membrane so as to commit injury ; and,
also, the blades themselves do not ap-
proximate by a considerable space. In
operating on a child some years ago,
the stone was so small that when I had
got it in the blades of the forceps it
slipped out again through the aperture,
and I was obliged eventually to take a
small pair of dressing forceps, the blades
of which fitted to each other, before I
could seize the stone so as to draw it
out, protracting the operation, but not
increasing the risk.*
Another inconvenience I have met
with has been when the stone was ex-
ceedingly soft, so that upon application
with moderate force of the forceps it
has crumbled down like half-dried mor-
tar, and I have been obliged to extract
it piece-meal afterwards, to use a scoop,
(an instrument for the purpose of
taking out small fragments of that kind,)
and afterwards to inject the bladder, so
as to get rid of finer portions. In a
case of this kind, the operator should
carefully search every portion of the
bladder, to ascertain whether it is com-
pletely emptied of the foreign matter.
In order to do this, having met with
difficulty in the case to which 1 have
alluded, I have had a sound made with
a rather bulbous extremity, by which I
can detect a fragment better than with
the common instrument. If you leave
a small fragment behind, the mischief
is, that it forms a nucleus for further
incrustation or deposit.
Another instance of difficulty which
occurred, not in my own practice, bat
in a case where I was asked to be pre-
sent at the operation, arose from the
stone being encysted. This has always
led me to examine a patient several
times before an operation, to be satisfied
that this circumstance did not exist
before I determined upon operating.
An elderly gentleman was the subject
of stone, the symptoms were not severe,
the stone was readily detected by in-
troducing a sound into the bladder, but
I learnt after the operation that it was
uniformly felt at the same spot, and in
consequence of this the detection of
the stone was easy. The operation was
decided upon, and when the forceps
were introduced through the wound into
the bladder, the extremity of the stone
could be felt, but it could not be grasped,
the greater part of it being enclosed in
a cyst, and presenting a small aperture
through which only a small portion of
the stone could be brought in contact
with the forceps. After trying various
unsuccessful means to get hold of it,
the operation was necessarily abandoned
without extracting the stone, because
great mischief would have ensued by
the further continuation of such at-
tempts."
The circumstance of always feeling
the stone in the same situation is not
conclusive evidence of its being en-
cysted. We lately had an old gentle-
man under our care in whom we always
ascertained the presence of a calculus
in the bas-fond of the bladder, on the
right side, immediately behind the pros-
tate. On the performance of the ope-
ration he was found to have several
calculi, not one of which was encysted.
4. Dangerous Consequences of the Opera-
tion.
Mr. Tyrrell's remarks on this head
are incomplete. After attending to hae-
morrhage, he sums up the causes of
death in this way.
" Another, and perhaps more remote
circumstance is, retention of urine, and
in some few instances, perhaps four or
five in my own practice, I have had, in
the course of the afternoon following
the operation, to pass in a catheter
* Since these remarks were made I
have operated on a young child, and
the stone, being very small, lodged be-
tween two folds of the bladder, from
whence I was obliged to move it by my
finger before I could seize it with for-
ceps. The child is quite well.
544 Pkriscopk ; or, Cihcomspkctivk Kkvikw. [Oct. 1
through the upper part of the wound,
to prevent the patient becoming dis-
turbed by the accumulation of water in
the bladder. If the stone is of mode-
rate size, from the cut being clean, and
no force used in the extraction, there is
no contusion, and the parts become
nearly agglutinated in a few hours by
the deposition of adhesive matter; but
still the irregularity produced in part of
the urethra prevents the urine from
taking its ordinary course. The same
operator to whom I just now alluded,
(Mr. Liston,) invariably after the ope-
ration passes a flexible gum tube into
the wound, and allows it to remain for
several hours, so that as the urine enters
the bladder it escapes by the tube. I
do not see much utility in this proceed-
ing if you watch the patient. If the
urine do not flow readily, no additional
disturbance is produced by introducing
a female or flexible catheter through
the upper part of the wound ; where-
as, I should think in some instances,
the presence of the extraneous matter
might induce suffering.
Further than this, We have inflamma-
tion attacking the cellular tissue about
the wound, and I believe it occurs most
frequently where there has been con-
siderable force or violence employed.
And, again, in a few instances, we have
peritonitis supervening."
As diffuse cellular inflammation is
decidedly the most frequent cause of
death after lithotomy, as it is an affec-
tion of which no very good description
has been given, and as many surgeons
and most pupils are but imperfectly
acquainted with its characters, pro-
gress, and treatment, it would probably
have been well had Mr. Tyrrell touched
on it more fully. It deserves more than
a mere mention.
Three other causes of death are not
hinted at.
The first is circumscribed abscess of
the pelvis. We have seen three in-
stances of death from this after litho-
tomy, and a lady in whom we divided
the spincter ani, for ulcer of the rectum,
died of it very lately.
The second is inflammation of the
vesical veins. We have once seen this
prove fatal, with purulent depositcs in
the joints, and several cases of the sort
have been published.
The third is collapse, without any
obvious organic lesion. We have not
seen such a case ourselves, but we
have heard of one. The patient was a
boy.
We merely enumerate the preceding
causes of death after the operation, in
order that Mr. Tyrrell's observations
may be rendered more complete. We
are aware that other serious effects, of
an indirect description, are observed in
some rare cases. But these are rather
consequences of an operation,than of the
operation. On the whole, it may be
said that the most pressing immediate
danger from the operation is haemor-
rhage?the most fatal consequence, cel-
lular inflammation. Haemorrhage must
always be regarded with a jealous eye
?not merely on its own account, but
for the disturbance to the cellular tissue
that it creates, and the greater tendency
remarked after it to diffuse or circum-
scribed cellular inflammation. Secon-
dary haemorrhage, we should observe,
has been witnessed, and has even, we
believe, proved fatal.
We cannot compliment the reporter
of the case on the manner in which he
has related it. The details are extremely
prolix, yet, withal, imperfect. The state
of the urine is very inadequately de-
scribed, and the composition of the
sabulous matter, as we observed, is not
hinted at. As the stone is stated to
have been lithic acid, we may suppose
that the " grit" was the same. The
reporter being a pupil, will, we have no
doubt, take the hint in good part.
III. Medullary Carcinoma.
Three cases of this affection are de-
tailed. The first is interesting on ac-
count of the extensive diffusion of the
disease, and its co-existence with mela-
noid and haematoid tubercles. The
second is interesting also, from the
tumor being developed subsequently to
repeated venaesection. The third case
is an instance of lupus in early life and
medullary carcinoma afterwards.
Case 1. All that it is necessary to state
183G] Medullary Carcinoma. 545
of the particulars of this case are the
following. The patient was 64 years
of age?the disease commenced as a
small, hard, moveable tumor under the
skin of the outer side of the forearm,
rather more than four years before his
death?the tumor fungated and bled?
two years after its commencement it
was removed by ligature?a fungoid
excrescence then sprang up in the cica-
trix?and finally various tumors ap-
peared on the body?nearly threemonths
before his death, he had hemiplegia of
the right side, which was relieved?a
fortnight before his death he had an
attack of complete insensibility and
paralysis. The principal interest be-
longing to the case is connected with
the universality of the disease, disclosed
upon dissection.
Dissection. The tumors described in
the narrative of the case, situated in
the subcutaneous cellular tissue, were
found to be encysted, and lobulated by
the presence of secondary cysts, as was
proved by section ; the contents were
of the consistence of cheese ; in some
the cyst was distinct, in others not;
and in some, vessels were very percep-
tible. They possessed very satisfacto-
rily the well-known characters described
by authors under the several terms
cephaloid, melanoid, and hsematoid tu-
bercles. For the most part each might
be classed under one of these heads;
though some sections appeared to par-
take of the character of each.
Head.?There were several tumors
in different parts of the brain, varying
in size from that of a pin's-head to that
of a large walnut, and they were all
surrounded by cysts more or less per-
fect. One of the largest was situated
in the left corpus striatum, projecting
into the lateral ventricle. Another oc-
cupied the greater part of the posterior
portion of the left thalamus opticus,
and extended into the posterior lobe of
the brain. These tumors were solid,
and firmer than the cerebral substance.
Upon section they presented the appear-
ance of a clot of blood, in part deprived
of its colouring principle ; some were
firm and striated, others were traversed
by vessels. Some, as the two following,
contained a fluid in the centre resem-
bling grumous blood ; viz., one in the
left middle, and one in the right poste-
rior lobe of the hemisphere: the sub-
stance of the brain contiguous to the
cysts was softened; in other parts it
was quite healthy.
Chest.?The large tumors under the
clavicles encroached very considerably
upon the chest: the osseous structure
of each second rib was destroyed, ex-
cepting their spinal extremities, where
a few scales of bone remained. Upon
making a longitudinal section in one of
these, a succession of smaller tumors
were exposed, of the mixed characters
of the cephaloid, heematoid, melanoid,
and white tubercle; here this appear-
ance was strongly marked. The sub-
stance of the upper part of the sternum
and the right clavicle were softened,
and infiltrated with a cheesy substance.
In the left pleura pulmonalis, at its
apex,was a cartilaginous deposite,about
two inches in length and half an inch
in thickness. In the muscular sub-
stance of the heart were three tumors
about the size of peas, and there was an
irregular one projecting into the cavity
of the right ventricle.
Abdomen.?Two medullary tumors,
the size of walnuts, were observed in
the spleen; several in the omentum,
and one, which was ulcerated, in the
mucous membrane of the ilium : the
left renal capsule was very much en-
larged, and converted into a soft me-
dullary substance; and there were two
small htematoid tumors in the right
kidney.
Case 2. A man, set. 52, admitted
June 20, 1834. Fifteen years ago, says
the report, having received an injury tov
the head, he was blooded three times
successively from one vein in the right
arm ; from this period he dates the for-
mation of a small subcutaneous tu-
mour at the bend of the elbow, which
remained stationary till within the last
four months, when it began suddenly
to enlarge, and has now acquired an
extraordinary bulk. It extends from
the insertion of the coraco-brachialis
muscle to the fossa below the bend of
the elbow-joint; its lateral dimensions
twenty-four inches, its form nearly
546 Periscope; or, Circumspective Review. [Oct.
spherical, and traversed by large veins,
having four or five discoloured undtila-
tory prominences. Some enlargement
and induration of parts in the axilla?
several small tumors felt in the subcu-
taneous cellular tissue of the abdominal
parietes. The patient had cough, spat
blood and muco-purulent matter, and
had pain in the chest. On the 26th he
had an apoplectic seizure. On the 27th
he died.
Dissection. A very large apoplectic
cyst in the left hemisphere of the brain
?the cerebral substance softened. A
curdy tuberculous deposite in various
parts of the lungs. The spleen much
enlarged, and converted into a semi-
fluid mass, of the colour and consis-
tence of grumous blood, mixed with
soft whitish tuberculous deposit.
The tumor itself seemed of uniform
lardaceous structure, little organized,
and of firm consistence. Several simi-
lar deposites were seated in the inter-
muscular spaces of the left arm.
Case 3. A gardener, aged 31, had
had lupus in early life, indeed until the
age of 20. Fourteen months prior to
his death, the cancerous disease com-
menced with an abrasion in the left
groin. Having thus commenced, it
extended, fungated, and finally acquired
such dimensions and characters as the
following:?it reached from about three
inches above Poupart's ligament to the
cleft of the nates; the centre gapes,
with broadly everted margin, forming
a large cavity ; the granulations have
a fungoid character, and are covered
with a sanious ichor, very copious and
fetid. He then died.
Dissection. The carcinomatous mass,
of an irregular circumference, entirely
filled the left iliac fossa. No vestige
of psoas or iliacus muscle could any
where be detected upon section. A
compact, tough, inorganized substance
of a yellow white colour presented it-
self, studded with tubercles of an oval
shape, varying in consistency, from a
hard cartilaginous matter to the sof-
tened state of a scrofulous tubercle.
The liver was soft and granulated, and
three tubercles of small size were found
in it, originating apparently at its sur-
face, and dipping down into its sub-
stance.
Mr. Travers makes some interesting
clinical remarks upon these cases.?
They are calculated to display the du-
bious and mysterious consanguinity be-
tween medullary carcinoma and scro-
fula.
IV. Mortification of Hernia.
A case of this sort is related at some
length. The patient died, but no ex-
amination was permitted, and so much
uncertainty hangs in consequence on
its actual characters and nature, that
we do not think it necessary to offer
the particulars.
A clinical lecture on the case was
given by Mr. Tyrrell, the surgeon under
whose care it was. The lecture, how-
ever, is chiefly calculated to explain
minute shades of symptoms or of treat-
ment, and to offer some ingenious con-
jectures on the actual cause of death.
The only passage we shall quote, is
one which bears on a question of gene-
ral interest and importance.
" I should say," says Mr. Tyrrell,
in remarking further upon this case,
"that there are no cases that present so
much variety as those of hernia. We
find regular symptoms noted in books
upon this subject, and usually detailed
in general lectures, which are to guide
the student when he enters upon piac-
tice, but if he relies upon these state-
ments, he will often find himself woe-
fully puzzled at the outset. It is not
unfrequently the case that, instead of
constipation, the bowels are relieved,
and freely relieved. A case of this kind
occurred some few years ago in this
hospital. A man advanced in life was
admitted, having a femoral hernia about
the size of an egg. The dresser at-
tempted its reduction by the use of the
warm bath and taxis, and rendered the
tumour less tense. The history of the
case was not sufficiently accurate for
us to know whether it had been re-
ducible or not, but the tumour being
reduced, the dresser administered some
purgative before I was sent to. I con-
sidered that the plan adopted had been
judicious, but that an injection should
1836] Statistics of Fever. 547
be used, and I desired, in case the in- ;
jection brought away faeces, that he
should have another purgative by the
mouth ; he did have it, and in the night ;
there were three or four copious evacu-
ations. The next morning, being anx-
ious about the case, I came down early,
and found the tumour in the same con-
dition, but rather more tender. I de-
cided, from the obscure history of the
case, upon operating, and found a per-
fect portion of the small intestine, the
entire calibre of which was girt by a
very firm stricture, and it was evident
that no feculent matter had passed the
stricture. All that we could say with
regard to the evacuation of the bowels
was, that the lower intestine had been
much loaded at the time the hernia was
strangulated, and that either the injec-
tion stimulated it to act, not once only,
but three or four times, or that the
sympathetic influence of the purgative
taken into the stomach had produced
an action on the lower part of the
bowels, which might be considered not
improbable. This is-one out of several
cases where I have known a free action
of the bowel, although there has been
perfect strangulation of the intestine,
even including the whole calibre of the
gut.
Again, you will find that patients
will evince but little tenderness of the
tumour ; sometimes the symptoms will
be such that your attention would
hardly be directed to the hernia, un-
less you knew that it was your duty,
under all circumstances, to make an
examination of a tumour of this kind.
There is sometimes so little pain in the
tumour,and such slightly marked symp-
toms, that the symptoms of strangu-
lated hernia are hardly present, or are
not so well marked as you find them
laid down in works on the subject, and
in general lectures.
I cannot put you too much on your
guard with respect to the examination
of the history of the tumour before you
decide on an operation of this kind, and
even before you attempt the application
of the taxis."
All this is very sensible, straight-for-
ward advice, and the young surgeon
should lay it carefully to heart. There
are, indeed, no cases in which caution
is more imperatively requisite than it is
in the diagnosis and treatment of her-
nia. No two cases of this disease are
exactly alike, and in no complaint has
ignorance or precipitancy committed
more grave or more palpable blunders.
This completes the- account of the
present number of the St. Thomas's
Hospital Reports.* We trust that both
the medical officers, the editor, and the
reporters, will continue to do their best
to maintain the scientific reputation of
a hospital which boasts of the names
of Cheselden, Mead, Cline, Crawford,
and others almost as illustrious as they.
We will do our best to disseminate the
facts they publish.
Belfast Fever Hospital.
Statistics of FEVEit.f
Dr. Mateer very properly observes, that
statistical investigations have begun to
attract attention, and to meet encou-
ragement. Many facts have been al-
ready made out by means of these ex-
tensive numerical data, and some errors
of long standing, and of wide prevalence,
have been dispelled.
Dr. Mateer is physician to the Bel-
fast Fever Hospital, and he has come
forward with the facts, which his situ-
ation and.his zeal have enabled him to
collect, and led him to publish. Every
hospital affords materials for similar
observation and similar collections of
results. Dr. Mateer makes the follow-
ing suggestion.
" In all such institutions, records
are kept of the age, trade, residence,
habits, &c., of the cases admitted into
them; and were these arranged in a
tabular form, much useful information
might be elicited from them. The task
of reduction could easily be committed
* Notices of the Reports on Rheu-
matic Pericarditis?Chronic Laryngitis
and Enuresis, will be found in other
parts of the Periscope of our present
number.
f Dublin Journal, September, 1836.
548 Periscopk; or, Circumspective Review. [Oct. 1
to intelligent patients, confined with
trifling ailments, under the superinten-
dance of their medical attendant. In
this way the present tables were pro-
cured. They detail all the particulars
regarding fever that could rightly be
ascertained, or were worth knowing:
Buch as, for example, the amount of
deaths and admissions, ratio of mor-
tality, number of cases from each street
or locality, of each trade, and the dura-
tion of illness both before and after ad-
mission."
We shall lay before our readers such
conclusions as seem to enlarge or cor-
rect our views of fever, omitting some
observations of a more speculative cha-*
racter on its nature, causes, or treat-
ment.
After remarking that the distinctions
of exciting and predisposing causes of
fever are not always tenable, Dr. M.
goes on to state, that contagion and
atmospheric influence may be classed
under the head of exciting causes. Epi-
demic fever is commonly attributed to
the operation and influence of altered
states of atmosphere. What those al-
tered states are, it is always difficult,
perhaps impossible, to say. They would
seem to be independent, or nearly so, of
the temperature of seasons. During
the eighteen years, ending May, 1835,
there were admitted into the fever hos-
pital 5078 cases during Summer; and
4771 in Winter; making a difference
of only 307 for such a lengthened pe-
riod. The following table of quarterly
admissions shows the number for each
season, and also the general mortality.
From May, 1818,
to May, 1835.
Admitted.
flH
Died.
General Mortality
for each Season.
1st quart. Summer,
2nd do. Autumn,
3d do. Winter,
4th do. Spring,
1191
1157
1081
1191
1405
1325
1278
1221
2596
2482
2359
2412
82
79
79
100
66
77
89
76
148
156
168
176
1 to I7f| for Summer.
1 to 15|| for Autumn.
1 to 14j'j for Winter.
1 to 13j5 for Spring.
Total Amount,
4620
5229
9848*
340
308
648
Dr. Mateer alludes to the operation
of contagion as a cause of fever, and
the table we shall next copy purposes
to afford an estimate of its effects.?
Our author notices, but not with ap-
probation, the disposition which exists
to discountenance the idea of contagion,
and to attribute to other influences,
what has been generally considered to
result from it.
" Any one, however, who has at-
tended hospitals, must have found that
such a supposition is quite groundless.
He will have observed patients being
brought in a short time after their
friends or acquaintances, or that they
are followed by one or more of the re-
maining members of the family to which
they belonged. A table has been drawn
up from the registry, with the design
of throwing light on this matter. In
this table are marked the number of
cases which may have had any com-
munication, either by residence in the
same house or by belonging to the same
family. It has been supposed that when
two or more individuals have been
brought from the same house or family,
the disease has originated in them from
contagion, there being communication
* This sum includes moribund cases and relapses, amounting to 252, which
are not included in the other calculations.
1836J Statistics of Fever, , 549
in these instances, and consequently ex-
posure to infection. The remainder are
set down as single cases, or those where
no contagion could be traced ; though
it is obvious, from the way in which
our calculations on this point are
framed, that in many of these this cause
may have been in operation, so that the
subjoined table does not adduce all the
truth in favour of contagion."
This argument is singularly weak,
and constitutes a direct petitio princi-
pii. If we are to admit that facility of
communication is sufficient proof of
contagion, then there is no epidemic
complaint which is not contagious.
Common catarrh must immediately be
regarded as a very contagious malady.
It is obvious that any general influence,
as that, for example, of the atmosphere,
"will be likely, cseteris paribus, to ope-
rate similarly on persons of similar con-
stitutions, placed under similar circum-
stances. If one member of a family is
attacked with fever, itself the produce
of certain atmospheric conditions, the
other members of that family are surely
more susceptible of the same morbid
action than the members of other fa-
milies, or solitary individuals would be.
The ground that Dr. Mateer treads on
is dangerous and hollow :?
Incedit per ignes
Suppositos cineri doloso.
We cannot, therefore, lay much stress
on the table we are about to insert.
The principal point which it seems to
establish, is the influence of variability
of season. The years 1826, 1828, and
1831, are stated to have been very
changeable, and we find that in these
the number of admissions is nearly dou-
ble that of the years preceding them.
This, however, is not the case with the
year 1828, while 1818, of which the
variability is not noticed, exceeded all
its successors in the number of its vic-
tims.
Date
from ? to
May ,, May
1818 ? 1819
1819 ? 1820
1820 ? 1821
1821 ? 1822
1822 ? 1823
1823 ? 1824
1824 ? 1825
1825 ? 1826
1826 ? 1827
1827 ? 1828
1828 ? 1829
1829 ? 1830
1830 ? 1831
1831 ? 1832
1832 1833
1833 ? 1834
1834 ? 1835
Total
?.S ?
I Ss
s s s
C3
^ O [i.
905
590
680
210
265
185
305
205
701
560
355
130
340
705
360
315
385
7246
^ .a
ai JZ
a s
?3
215
95
132
65
86
62
95
92
175
138
101
67
78
188
75
72
120
1856
o ? jj*
? 2 :5
g 2
(U O c3
? ? rt
g CL, rg
4^ 353 1258
6jV 90 680
5jf 47 727
3fj 49 259
3/, 40 305
3jx 36 211
315 103 408
2/o 107 312
4-2^s 157 858
3i's 196 856
318 126 481
2 25 215
4? 228 568
3'f 304 1009
4ff 177 537
4? 162 477
3j? 252 637
2342 9588
Speedy removal to hospitals is insis-
ted on. It would seem that the dan-
gcr of a fatal issue is in the ratio to the
length of time that the sick remain in
No. L. O ?
550 Pkriscopb; on, Circumspective Rkvikw. [Oct. 1
their houses previous to admission.
This, we say, would appear to be proved
by the following table.
to 2 2
e
3 3
a* a"
But, probably, a source of fallacy
sticks to this calculation. We must
recollect that only the very bad cases
are taken to a hospital late, while early
admissions must necessarily be more
indiscriminate. If a patient is kept at
home by his friends for the first week,
they are not likely to send him to hos-
pital in the second, if he has an appa-
rent chance of doing well. The late
cases are, therefore, more picked and
worse cases than the early ones, an
element to be received into our calcu-
lations.
The gross mortality of fever is natu-
rally a subject of rational curiosity. It
varies every year in the hospital, and it
varies out of it. Probably extensive
series of years would not display great
differences. The general mortality for
the eighteeen years ending May, 1835,
is 6/f^j per cent, or as 1 to 15^; de-
ducting moribund cases, it would be
only as 1 to
" The predisposing causes of fever
are various, such as age, trade, vicious
habits of living, innutritious diet, bad
ventilation. The most uniform results
obtained from any part of our calcula-
tions are those in reference to the in-
fluence that age has on the number and
mortality of fever cases. The follow-
ing condensed table will illustrate this.
It includes the period between Sept.
1817, and May, 1835, during which
there have been admitted at the age of
from?
Cases. Died.
1 to 5 .. 301 .. 12] Admitted, 5214 ;
5 to 10 .. 979 .. 13 I died, 151 ; being
10 to 15 .. 1709 .. 36 [ a mortality of
15 to 20 .. 2225 .. 90 J per cent.
20 to 25 .. 1384 .. 74") Admitted, 3747 ;
25 to 30 .. 1033 .. 81 I died 301 ; or a
30 to 35.. 677 .. 70 f mortality of 8-iffj
35 to 40 .. 553 .. 76 J per cent.
40 to 45 .. 418 .. 82") Admitted, 1043 ;
45 to 50 .. 302 .. 60 I died 216; or a
50 to 55 .. 188 .. 45 f mortalityof 20/535
55 to 60 .. 135 .. 29 J per cent.
60 to 65 .. 86 .. 31 ] Admitted, 171 ;
65 to 70 .. 36 .. 12 I died, 60; being
70 to 75 .. 25 .. 11 [ a mortality of
75 to 80 .. 24 .. 6 J 35^ per cent.
1836] Statistics of Fever. 551
Thus, the susceptibility to fever de-
creases with the increase of age, whilst
its comparative fatality increases. De-
crease of frequency and increase of
mortality are, therefore, in an inverse
ratio. This seems to be clearly made
out, and is a broad generalization of
some consequence.
Calculations have been made of the
number of fever cases admitted from
each lane, street, or "entry" in Bel-
fast. As a general rule, the streets in-
habited by the poorer classes were most
rife in the disease. But, what is curi-
ous, one street called Carrickliill, and
its continuation Millfield, with the ad-
joining lanes and entries, are found to
have supplied nearly three-fifths of the
whole amount; and yet these are by
no means the poorest or worst-venti-
lated parts of the town. The circum-
stance would seem to be caused by a
deficiency in the proper supply of water
to this district, from its occupying a
higher elevation than the source whence
the rest of the town is supplied. Wa-
ter is procured in other ways, but with
difficulty, and not in the quantities they
"Would otherwise have it. The conse-
quences are want of cleanliness and bad
sewerage, so that decayed animal and
vegetable matter of all kinds, not being
carried off by a current of water in the
usual way, accumulate, and so gene-
rate miasmata. The atmosphere of
such places may in this way be as much
contaminated as by improper ventila-
tion. Surely this offers the very strong-
est argument against Dr. Mateer's own
view of the extensive operation of con-
tagion. Why should this be all-pow-
erful in one particular locality ? Why
should it not do its worst, where
poverty and bad ventilation flourish?
Why, but that, on the large scale, other
causes of fever are far more potent than
contagion.
The influence of trade is next consi-
dered. It is difficult to arrive at accu-
rate conclusions on this head. For,
first, the ratio of numbers in any trade,
compared to the whole population,
should be made out; secondly, the ra-
tio of each to the other ; and, thirdly,
the results of private as well as of pub-
lic practice should be obtained. So far
as Dr. Mateer'a observations extend,
they are thus embodied:?In the 18
years already mentioned, there were
admitted into the Belfast Fever Hos-
pital?
Basket-makers
Brush-makers
Butchers. .
Blacksmiths
Blue-dyers .
Bricklayers .
Brass-founders
Bonnet-makers
Bleachers
Bakers .
Brick-makers
Bookbinders
Barbers . .
Cotton-spinner
Chandlers ,
Cutlers . ,
Coopers . .
Cotton-printers
Cotton-warpers
Carpenters . .
Curriers . . .
Constabulary-police
Chimney-sweepers
Carmen . .
Coach-makers
Copper-smiths
Clerks . .
Cabinet-makers
Dealers . .
Dress-makers
Flax-dressers
Flowerers
Founderers .
Gardeners .
Glovers . .
Glass-cutters
Glass-blowers
Hatters . .
Hosiers . .
Labourers .
Lightermen .
Lamp-lighters
Mariners
Musicians .
Mantua-makers
Mendicants
Nailers .
Painters .
Police-watchmen
Printers . . .
O o 2
2
15
39
51
26
53
7
30
35
35
25
12
6
464
10
8
59
49
10
76
12
9
8
34
10
12
31
12
218
58
26
88
10
11
7
6
10
5
11
878
13
4
131
23
11
16
2
22
9
18
552 Pkriscopk ; pit, Circumspkctivk Rkvikw. [Oct. 1
Pilots ....... 16
Paper-makers  4
Pork-cutters ...... 10
. Pensioners  8
Robers 31
Ragmen ....... 6
Shoemakers. 234
Servants ....... 1106
Sail-makers  4
Sempstresses ..... 116
Sawyers ....... 50
Stonecutters 25
Saddlers ....... 11
Stucco-workers .... 15
Students....... 14
Ship-carpenters .... 16
Schoolmasters 20
Staymakers  6
Tobacco-spinners .... 35
Tanners  6
Tailors ....... 79
Tinsmiths ...... 12
Upholsterers ..... 5
Weavers ....... 716
Whitesmiths ..... 10
Washerwomen ..... 40
Watch-makers  4
Turners  8
Various 66
Total amount of those occu- \
pied in trades ... J
A glance at the preceding table seems
to shew, that the persons most prone
to fever are placed under circumstances
almost diametrically opposed;?for, on
the one side, we see labourers, mari-
ners, and dealers, persons more or less
exposed to vicissitudes of temperature :
and, on the other, are ranged cotton-
spinners, shoemakers, sempstresses,and
weavers, individuals of sedentary habits,
and exposed to anxiety from fluctua-
tions in the labour market. Servants
suffer in great numbers. We scarcely
know in what category to place Irish
servants. Were they in London, we
should put them in the niche of idle,
gin-drinking people, who eat far more
than they work.
" Sex. would seem to predispose to
fever. Females are. more susceptible
of the disease than males. Thus, of the
total number admitted from May, 1818,
to May, 1835, inclusive, 5130 we find
to have been females, and 4458 males ;
being a superiority of the former over
the latter of 672. This may be ac-
counted for from the circumstance of
females being the more numerous part
of the population, to their more delicate
frame of body, which unfits them for
enduringhardships, and particularly also
to the custom (almost entirely confined
to females of the poorer classes) of go-
ing abroad barefooted, by which they
are exposed to the rigour and vicissi-
tudes of the seasons. But while the
susceptibility to fever is greater with
females, the mortality is found to have
been less with them than with the
males. Taking the general ratio of re-
coveries to deaths, in the admissions of
each sex for the period already referred
to, we find the recoveries with males to
be as 13|f to 1, while with females they
are as 1to 1."
The duration of fever is another in-
teresting subject of inquiry. Do exact
modern investigations support ancient
dogmatism on this head? Has fever
really a definite course?has it actually
critical days ? The following are the
contributions to a reply, supplied by the
industrious observation of our author.
Making allowance for the variations
occasioned by peculiarity of habit, mode
of living, and so forth, perhaps this
may be found an approximation to the
truth.
" From the general table annexed to
this paper, it will be found, that the
average time for each patient to remain
in the hospital under the disease is
about twenty-two days; and this cal-
culation is made from 11209 patients.
By the table that details the number of
cases admitted on each day of illness,
given in another place, it appears that
the mean number of days that patients
have been ill with fever before being
brought to the hospital, is about seven,
which, added to twenty-two, gives us
twenty-nine, as the average number of
days required by the disease to run its
course, from the time of attack, till
health is re-established.
During these twenty-nine days, the
complaint progresses for a definite pe-
riod, till a certain point of intensity is
attained, after which, if a fatal event
183G] Statistics of Fever. 553
have not taken place, the symptoms di-
minish in severity. This point, com-
monly termed crisis, occurs at some
fixed time. In one hundred consecu-
tive cases, carefully noted for this pur-
pose, it was found, on a mean calcu-
lation, that the fourteenth day was that
on which the crisis took place, or nearly
about one-half the time which, as we
have already seen, the whole course of
fever takes up in each individual. So
that it would seem to be proved by ex-
tensive observation, that the disease
takes up one-half of twenty-nine days
to progress, and the remainder to re-
trograde, or, in other words, that the
periods of increment and that of decre-
ment in the intensity of the symptoms
in fever, are equal and proportional.
The practical rule to be deduced from
this law is, that no patient ought to be
allowed to leave an hospital or his own
room, until at least a length of time
have elapsed, after a favourable change
has taken place, equivalent to that in-
tervening between this latter and the
commencement of fever. Otherwise a
relapse occurs, and nature, being foiled
in her work, all has to be done over
again ; and hence we find (as one of
our tables shews) that relapses happen
on those occasions only where this rule
has been broken through. Of 149 re-
lapses occurring from 1818 to 1828
(after which time no account was taken
of them in the registry), the mean du-
ration of the original attack is found
to be only twenty-two days, whereas,
in other instances, it is twenty-nine.
When recovery is taking place, there is
always felt, on the part of the patient,
an eagerness to get home, or to leave
his bed too soon, which the medical at-
tendants often find difficulty in repres-
sing."
This is very judicious advice, and, al-
though it is not to be taken au pied de
la lettre, there is no doubt of its being
a very excellent application of numeri-
cal results.
Passing over some general observa-
tions, to one of which, however, we
shall instantly revert, we insert the con-
cluding table. It renders the present
report complete, and a very praisewor-
thy report it is. Wc shall feel great
pleasure in throwing together the sta-
tistical records of practice that appear
from time to time in our contempora-
ries. Our object is to give as useful a
character as possible to clinical obser-
vations.
(See Table on the next page.)
We said that we would revert to one
point. It is the mode of exhibiting
wine in typhus. Much discretion is re-
quisite in its administration?discretion
both in timing and in measuring it.
Dr.Mateer has applied the fact, which he
seems in some degree to have establish-
ed, that about the tenth or eleventh day
the disease has reached its acme and
tends to decline, to the exhibition of
wine. Let him speak for himself:?
" The plan I have followed for some
time past is to keep from its use as long
as possible, and to begin with it only
when the pulse has sunk much, and
prostration of strength is very decided.
This for the most part happens on the
tenth or eleventh day of sickness, or on
the third or fourth after admission. The
next rule to be observed is, that when
we have commenced to use it, we are to
do so at first in small quantities, and
afterwards to increase it gradually till
the proper effect is produced. The dose
usually given at first to an adult is four
ounces. This may be daily increased
until, in the course of from three to four
days, it is found to amount to twelve
or fourteen ounces, which latter is, ge-
nerally speaking, the ' maximum' dose
that is advisable to be given. The day
on which this quantity falls due to be
given is commonly the critical day, or
the period when the febrile action has
reached its greatest intensity, and there-
after begins to decline. It is easy to
see whether the wine so administered
has had the proper effect, by the usual
favourable symptoms making their ap-
pearance. The patient falls into a sound
sleep or becomes quiet, and the coun-
tenance itself forms a sufficient index
of the favourable change that may have
taken place. When this has happened,
the large dose of wine is no longer to
be administered. It must be immediately
lessened, and that by tho same gradation
as we increased it, viz. two ounces daily,
till we come to the quantity we started
554 Periscope; ok, Circumspective Review. [Oct. 1
A TABLE,
Shewing the Number of Fever Patients admitted into the Belfast Fever Hospital, the Amount of Deaths, Moribund Cases, the
Ratio of Mortality, General and Annual, from Maij, 1817, to May, 1835; with an Account of the Expenditure for the
Ten Years, ending May, 1833.
DATE.
From ,, to
May ,, May
ADMITTED.
Males Fem. Total.
.'ales. Fem. Total
S3 5
O V " s
? " ?
E ^
?
<s.5u
tS Jj O
gO53.2
g?5"
0.2o
t 05
. A o o
"S-SS 3
d o$ *i3 S
? ? 5
Adm. Died
O'fjS" .
^ ?.? .3 B
a. sts a.-a
rf c ^
rt C3 " G co
o "a
<?S2
EXPENDITURE
FOR 10 YEARS.
ANNUAL AVE-
RAGE EXPEN-
DITURE FOR
1817 ? 1818
1818 ? 1819
1819 ? 1820
1820 ? 1821
1821 ? 1822
1822 ? 1823
1823 ? 1824
1824 ? 1825
1825 ? 1826
1826 ? 1827
1827 ? 1828
1828 ? 1829
1829 ? 1830
1830 ? 1831
1831 ? 1832
1832 ? 1833
1833 ? 1834
1834 ? 1835
520
311
368
124
148
115
182
143
391
299
219
103
258
479
268
229
301
738
369
359
135
157
96
226
169
467
357
262
102
310
530
269
248
336
1621
1258
680
727
259
305
211
408
312
858
656
481
205
568
1009
537
477
637
20
17
29
14
15
10
14
10
28
20
8
7
21
44
30
19
31
79
62
41
41
19
27
23
25
19
60
36
20
10
47
79
52
43
60
Total Amt. 4458 5130 11209 337 327 743 103
1 to 20
20
16
17-d
' <u
132
US
9?
^ c/l
16 ?
16-f
14 3
18
24*1
20.2
12 ?
12?
10
11
10
to 26
23
24
22-3
32
i3'i
9?
C/3
22 ?
28-2
19 ?
28^
25g
23.2
13 y
13?
14
14
11
44
147
1 5 S3 7 1
49
43
31
0
1
3
2 ?
Si
3 ?5
o P.
ci >1
a .is o
rt
0S5 C3
25
25
22
24 T3
23 $
23 g
21 ?
21 Jj
25 -9
24 I
22 15
25 ?
25 .2
25 ?
22 ?
20
20
21
?. s.
1247 11 Oi
1265 9 0
1108 8 8
1546 15 'o
1692 10 4
1373 14 3?
1410 7 7
1639 1 lli
1604 14 5i
1467 11 H
Wine,
?. s. d.
85 16 4
Medicines,
185 2 0?
Food,
650 0 7
Furniture,
Clothing, &c.
92 10 0i
Coals, Soap,
and Candles,
90 2 7
Salaries,
150 16 3?
Various,
153 5 11
ISffi 149 | 3
22T%?3 14077 12 2^| 1407 15 3^
1836] Statistics of the London Hospital. 555
from, when after a day or two its use
may be dispensed with, and a pint of
beer given for some time instead. This
plan of treatment, together with the
regular exhibition of purgatives or lax-
atives, as occasion would require, and
sometimes of tonics during convales-
cence, is "the only one that has been
found necessary to adopt for the cure
of typhus."
Of course observations of this sort
must always be received with some re-
serve, or, rather, they must not be made
to bear more than they will fairly carry.
When we come to individual cases, we
must be guided by their particular cir-
cumstances. The recollection of a gene-
ral principleisveryserviceable,but it must
not be rigidly applied to particulars,
because general principles are, for the
most part, averages of individual facts
that differ materially in many of their
features.
London Hospital.
Statistics of this Hospital, with
Remarks on the Law of Sick-
ness. By T. R. Edmonds, Esq.
B.A.
The present number contains some
statistical reports from the Birming-
ham Infirmaries, and from the Belfast
Fever Hospital. Mr. Edmonds, of Tri-
nity College, Cambridge, has published
a short paper under the title we have
copied in a late number of the Lancet.*
The paper itself is very valuable, and
deserves a more permanent place before
the profession, than the rapid revolu-
tions of a weekly journal will probably
afford it. We will abridge it as much
as is consistent with the preservation
?f its utility.
Mr. Edmonds very properly insists
on the necessity of being acquainted
with the general laws of mortality, in
order that we may appreciate the effects
of remedies. He volunteers a caution to
the profession, which, although very
proper, appears to involve a slight de-
gree of fallacy.
" The merit of discovering some pe-
culiarly efficacious treatment of a par-
ticular malady, is frequently claimed by
different medical men, on the ground
of the mortality among their patients
being unusually low. All mention of
the ages of their patients is omitted,
and no suspicion appears to be enter-
tained of the fact that, under the same
medical treatment, a difference of 23
years in the ages of two classes of pa-
tients, will cause a doubling of the
mortality. When the ages of the pa-
tients are unknown, the diminished du-
ration of sickness, also, is no ground
for presuming on any superiority of
treatment. There exists satisfactory
(though indirect) evidence, that the
mean duration of an attack of sickness
is equally dependent on the ages of the
patients."
No doubt that voluntary or uncon-
scious ignorance of the general laws of
disease, has led to much vaunting, if
not to actual cliarlatanerie, with respect
to the action of particular modes of
treatment in prevailing maladies. Dur-
ing the existence of the cholera, for
example, while many people were boast-
ing of their almost uniform success,
the statistical results, such as could be
got, shewed the general ratio of mor-
tality and recoveries almost unaffected.
But we do not think that well-in-
formed medical men have committed
the grievous blunder charged on them
by Mr. Edmonds, and failed to make
allowance for difference of age. It
needed not life-assurance offices to tell
us, nor profound equations to demon-
strate, that age is the great regulator of
mortality. From the very first year of
medical observation, empirical as it
was, to this, physicians have recogniz-
ed the obvious truth, and universally
acted on it. Statistics may give in-
creased precision to ideas founded on
desultory observations, but they can be
hardly expected to do more.
If Mr. Edmonds' strange assump-
tion of gross carelessness or ignorance
on the part of the profession is un-
founded, the conclusion which springs
from it falls to the ground. When
Sept. 3, 1836.
556 Pkhiscope; or, Gircumspkctivk Rkvikw. [Oct. 1
medical men claim success from a par-
ticular mode of treatment, they are not
such asses as to confound all ages, or
to think that because bleeding cures a ro-
bust man of a pleurisy, it will therefore
cure a feeble octogenarian. A scientific
physician or surgeon who recommends
a peculiar remedy in a peculiar case,
takes care to point out with infinitely
more precision than Mr. Edmonds
seems to dream of, not the age merely,
but the habit and the constitutional
state of the patient that it is adapted
for.
Equally strange with the preceding
accusation is the statement which fol-
lows :?that " no suspicion appears to
be entertained of the fact, that, under
the same medical treatment, a difference
of 23 years in the ages of two classes
of patients, will cause a doubling of
the mortality." No suspicion appears
to be entertained by Mr. Edmonds of
his committing a very great absurdity.
Where is the medical man who would
employ the same medical treatment in
two classes of patients, one 23 years
older than the other ? The exact ratio
of mortality with advancing age is, no
doubt, imperfectly known, but no phy-
sician in his senses would imagine
that such a difference of years could
fail to exert a very great influence on
the efficiency of medicine. Statistics
have not been sufficiently cultivated,
but such a thing as common sense has
not been quite absent from the minds
of the profession, and they are not al-
together so ignorant as Mr. Edmonds
supposes.
His next assertion is as startling a3
those we leave behind, and if statistics
lead to this, they will revolutionize not
only opinions but facts.
" Between the ages of 15 and GO years,
I believe it to be a fact sufficiently well
established, that for every death two
years of sickness (nearly) have been
suffered."
Mr. Edmonds must mean that the
above is the average. He cannot wish
his words to be literally taken. Yet
the expression is loose for a statistician.
He proceeds:?
" If, as is commonly the fact,
the annual deaths between these ages
amount to per cent., there will be
constantly sick 3 per cent, of this part of
the population. Respecting the num-
ber of cases of sickness from which the
numbers constantly sick have been de-
rived, we have very little direct infor-
mation. If the mean duration of an
attack of sickness be assumed to be
36? days, or the tenth part of a year,
then 30 per cent, of this population
are yearly attacked by sickness. In
the British Medical Almanac for 1836,
Mr. Farr has shewn the above number
of days to represent very nearly the
usual duration of an attack of sickness
in the hospitals of England. Out of
30 cases of sickness, there will then be
l? deaths, being 5 per cent., or 1 out
of 20. In the hospitals of London the
deaths to cases commonly amount to
12 per cent, among the in-patients, in
the physician's wards 24 per cent., and
the surgeon's wards 8 per cent. If the
out-patients had been included, the
proportion of deaths to the total cases
would probably not have materially
differed from the above-mentioned pro-
portion of one case in twenty."
The succeeding suggestions should
be carefully attended to by those
who wish to observe or to state the
results of their observations.
" In order to determine the sanitary
state of any population for one year,
the elements to be observed are,?the
number living, the number dying, the
number attacked by sickness, and the
mean number constantly sick, all dis-
tinguished according to age. When the
third and fourth numbers have been ob-
served, the mean duration of an attack
of sickness at each age is known. A
definite result cannot be obtained, un-
less two, at least, of the above four
elements be simultaneously observed.
It is not, however, indispensably ne-
cessary that more than two of these
elements should be observed at one
time. If, for example, from one ob-
servation we obtain the relation between
the living and the dying,?from ano-
ther, the relation between the dying
and attacks of sickness,?and from a
third observation, the relation between
the numbers attacked and the numbers
constantly sick, we shall then have all
1836] Statistics of the London Hospital. 557
that is required to determine the mutual
relation of all the four elements."
Hitherto we have had no reports so
complete as to embrace all these ele-
ments of calculation. Some have been
defective in one way, some in another,
yet, Mr. Edmonds remarks, that the
few observations that have been made
from a complete chain of connexion
between the four elements.
" There have been published from
many hospitals, reports of the total
number of cases admitted, of the total
number of deaths in hospital, and of
the mean number constantly sick. But
this information is generally of little
value, because no report is made of the
ages of the patients, of the numbers
discharged uncured, or of the propor-
tion of mild and severe cases of disease.
The distinction of acute and chronic
disease is also important; the latter
ought to be referred to the age and time
from the first attack. The first valu-
able statement on the influence of age
in determining the mortality from a
given number of cases of sickness, pro-
ceeded from the London Fever Hospital.
This statement was inserted in The
Lancet of Feb. 27, 1836, not having,
as I believe, before appeared in any
work addressed to the medical profes-
sion. Dr. Southwood Smith, the au-
thor of this statement, has omitted to
state the facts on which his results are
founded. These results are, however,
perfectly satisfactory, the defect in the
detailed statement of facts being, pro-
bably, of the least possible importance
in the case of a Fever Hospital. The
proportion of " discharged uncured"
of chronic cases, and of mild disease,
are here all at their minima. Accord-
ing to Dr. Smith's observation, the
deaths out of a given number of cases
?f sickness between the ages of 20 and
60, increase regularly 34 per cent, for
every advance of 10 years in age. The
present observation made at the London
Hospital, agrees in the indication of
the same progressive increase of morta-
lity according to age, in the physician's
wards, and also in the surgeon's wards.
The observation of Dr. Smith is found-
ed on 6,000 cases of fever occurring
during the 10 years ending with 1833.
The London Hospital observation is
founded upon 13,870 cases (and 1,622
deaths) occurring during the six years
ending with 1833."
Mr. Edmonds proceeds to lay before
his readers three tables of facts observed
at the London Hospital. Those facts
have been obtained from a Parliamen-
tary Return lately published.
"In this return the facts are given
for each year, in the same form as they
are now presented in the aggregate for
six years. The patients are distri-
buted in decennial intervals of age ; the
males are distinguished from females;
and these are again distinguished into
physician's and surgeon's cases. The
cases admitted at each age are disposed
of under four heads, cured, relieved,
irregularly discharged, and dead in hos-
pital. In the table of results I have
included the ' Relieved' and the ' Irre-
gularly Discharged' under one head,
that of ' Discharged Uncured.' If there
had been monthly (or even quarterly)
enumerations, according to age of the
patients remaining in hospital, the infor-
mation would have been complete, and
we should have known the mean dura-
tion of a case of sickness at each age.
It is not yet too late to make such an
observation, for the proportion admitted
at each age appears to be of a perma-
nent nature, and the proportions re-
maining in hospital at each age may be
presumed to be equally permanent. A
very small number of such enumera-
tions would, probably, lead to a valu-
able approximation to the mean and
almost permanent form in which the
patients remaining in hospital are dis-
tributed according to age. (The mean
number of patients remaining in the
London Hospital is 300, without dis-
tinction of age or class)."
558 Periscope j ou5 Circumspective Review. [Oct. 1
Tables, showing, for each of eight intervals of age, the numbers Admitted, Cured,
Discharged, and Dead, in the London Hospital, during six years, 1828-33.
(1.)?Physician's Wards.
Age.
0?10
10 ? 20
20 ? 30
30 ?? 40
40 ? 50
50 ? 60
GO ? 70
Above 70
All ages
20 ? 50
MALES.
O
14
270
629
498
301
149
43
17
1921
1428
Ph
2
42
133
138
111
40
14
2
482
382
182
135
3
47
180
176
157
98
36
14
711
513
H O
20
381
997
861
600
307
95
35
3296
2458
FEMALES.
U
20
280
392
181
101
44
7
7
1032
674
78
115
72
49
24
7
3
353
236
111
73
275
200
E-1 O
33
416
622
330
222
95
28
? 16
1771
1183
Both Sexes.
Cases.
Death.
53
797
1619
1200
822
402
123
51
5067
3641
9
80
259
242
212
120
44
20
986
713
(2).?Surgeon's Wards.
Age.
0?10
10 ? 20
20 ? 30
30 ? 40
40 ? 50
50 ? 60
60 ? 70
Above 70
All Ages
20 ? 50
MALES.
V
218
729
862
899
566
362
159
63
3858
2327
97
254
348
308
196
116
52
24
1395
852
24
60
158
105
58
28
20
6
459
321
a e- o
19
36
73
73
94
64
32
23
358
1079
1441
1385
914
570
263
116
I
240 3740
414 6126
FEMALES.
u
223
302
377
268
198
136
67
40
1611
843
Pi
76
125
164
100
73
38
23
13
612
337
43
34
59
46
30
8
6
6
232
135
222
73
H O
412
477
623
444
321
201
119
80
2677
1388
Both Sexes.
Cases.
770
1556
2064
1829
1235
771
382
196
8803
5128
Death.
89
52
96
143
114
83
55
44
636
313
18.J6J Statistics of the London Hosjntaf. 559
(3.)?Table showing the results of the preceding tables, assuming the number of
cases admitted at each decennial interval of age to be 100.
Age.
Physician's Wards,
Males.
O
o
fcfia;
X 3
o
CO
5
J3
y c
p
Physician's Wards,
Females.
u
1 c
5 3
Surgeon's Wards,
Males.
o
tco
?r a>
C3 is
Surgeon's Wards,
Females.
o
toy.
0?10
lo ? 20
20 ? 30
30 ? 40
-10 ? 50
50 ? GO
GO ? 70
Above 70
70-0
70-9
63-1
57-8
50-2
48-5
45-3
48-6
15-0
16-8
18-8
21-8
23-6
19-6
16-8
11-4
15-0
12-3
18-1
20-4
26
31-9
37-9
40-0
GO ? 6
67-3
G3-0
53-4
45-5
46-3
25-0
43-8
21-2
24-8
24-3
27-1
29-7
30-5
46-4
18-7
18-2
7-9
12-7
19-5
24-8
23
28 -6
37
60-9
67-G
59-8
64-9
G1 ? 9
63-5
60-5
54-3
33-8
29-1
35-1
29-8
27-8
25-3
27-3
25-9
5-3
3-3
5-1
5-3
10-3
11-2
12
19-8
54-1
63-3
60-5
GO *4
61-7
67-7
56-3
50-0
28-9
33.4
35-8
32-8
32-1
22-9
24-4
23-8
17-0
3-3
3-7
6-8
6-2
9-4
19-3
26
All ages
20 ? 5o
58-3
58-1
20-1
21-0
21 -6
20-9
58-3
57-0
26-2
2G-1
15
16-9
63-0
62-2
30-2
31-4
6
6-4
GO-2
60-7
31-5
34-0
8-3
5-3
On casting the eye over the preceding
tables, we are struck by one or two
unexpected circumstances. In the first
place, the number of female patients
and of deaths below the tenth year of
age, exceeds that of the males. Thus
We find in the physician's wards 38
females admitted and 6 deaths?and
only 20 males admitted and 3 deaths.
In the surgeon's wards there are ad-
mitted 412 females, and 70 deaths?
and 358 males and 19 deaths. These
facts are opposed to some reports from
the Pennsylvania Alms-houses, which
"We published in our last number, and
We therefore direct attention to the cir-
cumstance. After the age of 20, the
mortality amongst the males materially
pxceeds that among the females; but it
is singular that the excess is greater in
the patients of the physicians than in
those of the surgeons. This fact is in
apparent contradiction to those who
have imputed the mortality of males to
their greater exposure to accidents.
Exposure, indeed, tells in more ways
than by leading to a broken leg, and
probably the solution may remain as
applicable and as accurate as ever.
" From the table of results it appears,
that out of 100 male patients admitted
into the physician's wards, between the
ages of 20 and 50, there die, in hos-
pital, 20.9. It cannot hence be con-
cluded (as is often done) that only
20.9 die out of 100 cases admitted, be-
cause it would then follow that 79.1
were cured, whilst the true statement
is, that there were only 58.1 cures cor-
responding to 20.9 deaths. The num-
ber ' discharged uncured' amount com-
monly to one-fourth part of the cases
admitted. Some of these cases will
terminate in recovery, and some in
death. Assuming that all cases termi-
nate either in recovery or in death, I
have adopted the following principle for
distributing the ' discharged uncured*
into these two classes :?Suppose out
of 15 cases of sickness, the report to
be made, that eight are cured, two
dead, and five discharged uncured. I
have then assumed that out of the five
cases discharged uncured, four termi-
nate in cure and one in death. The
cases whose termination is known, are
in the proportion of four recoveries to
one death, and we have no ground for
expecting a different result from the
cases whose termination is unknown.
The following table has been calculated
on this principle, by adding to the
deaths in hospital the proportion of
deaths due to the numbers discharged
5(30 Periscope; or, Circumspective Review. [Oct. 1
uncured. The general effect of this
correction is to increase the apparent
mortality 30 per cent, at each age. I
have, however, given the mean results
independent of this correction, for the
sake of comparing them with results
obtained in a similar manner from the
London Fever Hospital and from the
Marylebone Infirmary. In these latter
observations the numbers discharged
uncured have not been distinguished.
(4.)?Table showing, at each age, the proportion of Deaths in Hospital, corrected for the
Numbers discharged Uncured; also shoioing their Proportions without Correction,
compared with the experience of the Fever Hospital, and of the Marylebone Infir-
mary.
Age.
0?10
10 ? 20
20 ? 30
30 ? 40
40 ? 50
50 ? 60
CO ? 70
Above 70
London Hospital.?Including the presumed
Death of Discharged Uncured.
Physn's Wards
Males. Fern.
17-6
14-8
22-3
26-1
34-3
39-7
45-6
45-1
23-1
10-5
16-8
26-7
35-3
33-4
53-3
46-1
Surgn's Wards
Males. Fem.
8-0
4-7
7-8
7-5
14-3
15-0
16-8
26-7
23-9
5-0
5-8
10-1
9-1
12-2
25-5
34-4
Both Sexes.
Phys. Sur;
20-4
12-7
19-6
26-4
34-8
3G-6
49-4
45-6
16-0
4-9
6-8
8-8
11-7
13-6
21-2
30-5
Deaths in Hospital.
London Hosp.
Both Sexes.
Phys. Surg
16-6
10-1
15-4
20-0
25-5
27-5
33-2
38-8
11-2
3-4
4-4
6-0
8-3
10-3
15-8
23-0
Mary-
lebone
Infirm-
ary.
12-2
4 ? G
15-3
21 -5
21-7
32-3
2G-5
49-0
London
Fever
Hospit.
9*9
14-3
19-5
26-2
35-5
All ages
20 ? 50
27-0
26-5
21-0
22-9
9-7
9-3
12-1
8-0
24-0
24-7
10-9
8-7
18-6
18-9
7-5
5-8
18-7
19-2
It is worthy of remark that the ab-
solute mortality at any age (from 10 to
60) differs in a very inconsiderable de-
gree among the three classes of pati-
ents?in the Fever Hospital, in Maryle-
bone Infirmary, and in the physician's
wards of the London Hospital. With
respect to the relative mortality, taking
the experience of the Fever Hospital as
the correct standard, the rate of in-
crease is generally too high in the Ma-
rylebone Infirmary when it is too low
in the London Hospital, and reversely.
Among the male patients of the London
Hospital, the mortality increases less
than 34 per cent, for every difference of
10 years in age: among the female
patients the reverse is the case. This
disagreement can hardly be attributed
to any thing but a difference in the
causes of admission; for there is no
reason to suppose that the relative mor-
tality of males differs from that of fe-
males in the total population."
Mr. Edmonds makes some further
observations on a mathematical formula
for the rate of mortality. We leave
this, however, to the Insurance Offices.
Mr. Edmonds deserves much praise for
bringing the tables we have copied be-
fore the professional public, and al-
though we questioned some of his
positions at slarting, we are very sen-
sible of the credit due to him for his
exactness and ingenuity. We trust
that Mr. Edmonds will persevere with
these researches, and we shall feel great
pleasure in directing, at all times, par-
ticular attention to them.
Pennsylvania Hospital.
Report of Cases treated in tub
Surgical Wards. ByT. S. Kirk-
bride, M.D. Late Resident Physi-
cian.
This Report is contained in one of the
1836] Pennsylvania Hospital Report. 561
numbers of our able contemporary?
the American Journal of the Medical
Sciences.* We shall select some of
the more interesting or instructive cases
"which it contains.
1. Amputation of the Hand by Machi-
nery?no Ligatures required.
Robert R. set. 30, a healthy boy,
while attending to some machinery, had
his left hand entangled in it. The
hand was, in consequence, torn off,
"with a small transverse portion of the
lower end of the radius; the muscles
"Were ruptured about four inches above
thewrist, and drawn out, still remaining
attached by their tendons to the hand.
He was brought to the hospital the
same evening. There was very little
hsemorrhage, and no vessel requiring
the application of a ligature. As
nearly enough skin remained to form
a flap, it was gently drawn over the
stump, and dressed as usual after an
amputation. A little fever followed,
with slight sloughing of the integu-
ments over the stump, and a small col-
lection of pus above the wrist. With
these exceptions he did well.
2. Mode of Applying the Ligature for
Aneurism by Anastomosis.
Our readers may remember that, in
the latter part of 1829, a paper was
published by Sir Benjamin Brodie in
the Medico-Chirurgical Transactions,
?n the mode of tying those erectile
tumors that sometimes occur on the
surface of the body. Sir B. Brodie's
plan consisted in passing two long
needles beneath the tumor, crossing
them at a right angle. Under the
needles the tumor was then strangu-
lated by ligatu res.
It appears that a similar proceeding
"was adopted in January 1829, by Dr.
Barton of Philadelphia. He was of
course ignorant of what Sir B. Brodie
had done, and the coincidence is amus-
mg. This may serve to introduce us
to the following case, in which Dr,
Barton operated.
Case. A healthy child, nine months
old, was taken to the hospital, on the
3rd of December, 1834, on account of
an aneurysm by anastomosis, which
had existed since birth on the left side
of the cheek, near the angle of the
mouth. It had attained the size of a
small nutmeg. The ligature was ap-
plied in the following manner:?A
common hair-lip pin was introduced
through the integuments under the
tumor, so that about one-half of its
length projected beyond the margins ;
by this the tumor was elevated, and a
second pin introduced under the first,
and at right angles to it. A strong
ligature was then applied around the
base of the tumor, below the points
of the pins, as firmly as possible. The
child did not appear to suffer severe
pain, except at the moment of tighten-
ing the ligature. On the 6th the liga-
ture was removed, and the slough sepa-
rated on the 9th, leaving an ulcerated
surface, three-fourths of an inch in
diameter, and of healthy appearance.
Simple dressings were applied; the
granulations occasionally touched with
the argent, nit. and on the 3d of Janu-
ary, 1835, the parts had cicatrized com-
pletely.
I
3. Cases of Fracture of the Patella.
No. 1. Jane M. ret. 29, admitted Oc-
tober 16th, 1833. In falling backwards,
and making an effort to save herself,
fractured the right patella. Discharged
December 2d. Ligament rather more
than one-fourth of an inch. She ex-
perienced much difficulty in bearing a
tight bandage.
No. 2. John M. set. 61, admitted
April 5th, 1834. Fracture from a fall
with the knee upon the edge of a curb-
stone. Entered the hospital twenty
hours after the accident, withj great
swelling and tenderness of the part;
crepitation could still be detected, and
a separation of the fragments. The
limb was elevated, and leeches and cold
applied to the knee. He could not
bear a bandage till the 27th. Dis-
charged June 28th. No appreciable
separation of the fragments; union
perfect; upper part slightly depressed.
No. 3. Jane M. (No. 1,) with frac-
For August, 1835.
562 Periscope; or, Circumspective Review. [Oct. 1
ture of the other patella. Admitted
April 20th, 1834. Accident produced
as in the previous instance, in attempt-
ing to keep herself from falling, and
making great exertion with the sound
limb to save that which had been be-
fore injured. Discharged June 28th.
Union ligamentous, one-eighth of an
inch separation ; the upper fragment a
little more depressed than the inferior
one.
No. 4. Jane E. ast. 52, admitted
January 21st, 1835. Produced by a
fall upon the knee, fracture transverse,
and a smaller fragment half an inch
wide separated from the upper portion.
Discharged March 21st. No separation
can be detected between the upper and
lower portions of the patella, except at
the point where the small fragment ex-
isted, and here a line is detected ; this
last being a little depressed below the
others.
No. 5. John M. set. 32, admitted
January 9, 1835. Fracture induced by
muscular exertion. He was exceedingly
restless, had mania a potu, and loosened
the apparatus repeatedly; notwith-
standing which, union took place, with
a separation of less than one-fourth of
an inch. He insisted on leaving the
hospital, contrary to the1 wishes of his
attendants, on the 14th of March.
After his return home, it appears he
commenced bending his leg at once, and
met with some accident, by which the
union between the fragments was en-
tirely broken up, and they are now
between two and three inches apart,
without any intervening ligament. He
is again in the hospital for the purpose
of having something done for his relief.
No. 6. This is a case of compound
fracture, which was admitted on the
25th of March, 1835, and still con-
tinues in the hospital under treatment.
If any union has taken place, (June
6tli,) it is very slight.
" The above six cases of fracture of
the patella are all that occurred to the
writer during a residence of two years
in the Pennsylvania Hospital, and were
all he believes that were received during
that period. The comparative fre-
quency of the accident appears to have
been greater than has usually occurred
in that institution, as many months not
unfrequently elapse without there being
a single specimen in the wards, where
cases of fracture are always numerous.
During the two years ending in April,
1835, two hundred and forty cases of
fracture were under treatment, of these
six were of the patella, or one in forty.
Of these six, two occurred in the same
individual; three were in males and
three in females. Three were produced
by muscular action alone, two from a
fall upon the part, and that of com-
pound fracture by a piece of rock
thrown against the knee from an ex-
plosion."
Blockley Hospital.
Dr. Gerhard's Reports on
Pneumothorax.
Dr. Gerhard, physician to the Blockley
Hospital, has related two cases of pneu-
mothorax in the number of our con-
temporary, for February, of the pre-
sent year. He has related two, but
he promises three more. Indeed the
report appears to have been suddenly
cut short, for he commences by ob-
serving :?
" The following cases of Pneumo-
thorax are five in number; four of them
were treated and observed at the Penn-
sylvania Hospital, and one at the
Blockley Hospital, attached to the Phi-
ladelphia Alms-house. The cases ob-
served at the Pennsylvania Hospital
proved fatal; three of these patients
died of the immediate effects of the
perforation, one recovered from it en-
tirely, and died of a secondary pleurisy,
which supervened about a year after his
discharge from the Hospital. The last
patient is still under treatment. The
cases afford illustrations of most varie-
ties of the lesion in question."
We shall give a brief sketch of the
two, and detail the remainder when
they come to hand.
Case 1. Phthisis of eighteen months'
standing?Tuberculous Cavities in the left
Lung?Perforation occurring at the
1836] Blockley Hospital Report. 563
upper portion of the right Pleura?Ex-
treme Dyspnoea?Death an hour after the
perforation?Pleura distended with in-
odorous Gas, and slightly reddened around
the perforation.
A seaman, Bet. 26, admitted Jan. 14,
1835.
In January, 1834, he was first af-
fected with cough and pain in the chest.
In March or April, he was seized with
haemoptysis, which subsequently con-
tinued, more or less.
On admission he had cough?whitish
expectoration with streaks of blood?
and many suspicious phthisical symp-
toms. These gradually advanced, and
in March, the anterior part of the left
side yielded a perfectly flat sound on
percussion, and there was a strong
crackling during the inspiration ; when
he coughed this was replaced by gurg-
ling. The phthisis went on. Suddenly,
m the evening of the 5th of July, Dr.
Gerhard was summoned to him. He
found him sitting in bed, suffering great
dyspnoea, and complaining of intense
pain in the right side. The respiration
had become more feeble, but the per-
cussion was not materially altered. A
mustard pediluvium, a sinapism to the
back, and cups to the right side did not
relieve the dyspnoea. The respiration
Was afterwards scarcely heard in the
nght side, while the sound on percussion
became more clear, and the intercostal
spaces were distended. The counte-
nance was at first extremely livid, and
finally pale and anxious. Death oc-
curred about an hour after the beginning
?f the dyspnoea.
Thus, in an advanced stage of phthisis,
the patient was suddenly attacked with
acute pain in one side, attended with
feeble respiration, and increased reso-
nance on percussion. What could this
be but pneumothorax ?
Dissection fifteen hours after death.
_ The lymphatic glands on the left
side of the neck, from the clavicle
to the mastoid process, varied in size
from that of a small almond to a large
hen's egg. Their substance was con-
verted into a white cheesy matter,
varying in consistence from that of hard
cheese to a soft creamy pulp ; the cyst
surrounding them was dense and of a
white fibrous aspect. The left side of
the thorax was contracted ; the inter-
costal spaces more depressed than usual.
The right side was evidently distended,
and the intercostal spaces strongly pro-
truded ; the sound of percussion upon
this side of the chest was tympanitic
throughout its whole extent.
The right pleura was opened and
found distended with air. The lung
was reduced to about one-fourth its
natural size, and was in contact with
the spine. The pleura covering it pre-
sented some slight cellular adhesions at
the summit; about an inch and a half
below the top of the lung, at the part
corresponding to the upper portion of
the axilla, there was a little depression,
of a rounded form, about half an inch
in diameter, and of a dirty yellow
colour ; the centre of this depression
offered a rounded perforation nearly two
lines broad. When air was forced into
the trachea, it was expelled in a stream
from the perforation which communi-
cated with a tuberculous cavity of the
size of a large hazel-nut, immediately
beneath the pleura, which formed
a part of its walls. It communicated
freely with a bronchial tube, and con-
tained some softened yellow tuberculous
matter. A few yellow tubercles and
granulations were contained in the
upper part of the lung, which was
throughout permeable to the air. A
few ounces of limpid serum were found
at the base of the pleura, which was
brightly injected round the perforation.
The left lung was extensively tuber-
culated, with cavities in the upper lobe.
The pleura were universally adherent.
The rapidity of the march of the
fatal symptoms is not difficult of ex-
planation. One lung was so tuber-
culated as to be inefficient for the pur-
poses of respiration. The pneumo-
thorax put the other hors-de-combat.
Still the progress of the case was rapid.
The case shews well that pneumo-
thorax usually occurs in an early stage
of tuberculated lung. In the latter
stages, repeated pleurisies have usually
produced more or less perfect oblitera-
tion of the pleural cavity. If before
this has taken place, a small tuberculous
cavity near the surface of the lung hap-
564 Pkiuscopk; or, Circixmspkctivk Rrvikw. [Oct. 1
pens to give way, pneumothorax is the
consequence.
Case 2. Phthisis of eight months'
duration?Tubercles confined to the sum-
mit of the lungs?Sudden pain occurring
while in bed in the right side of the chest
?Distention of that side ; loud resonance
on percussion ; amphoric respiration and
metallic tinkling ; decubitus on the side
not affected?Death eleven days after
perforation.
A cupper, set. 27, admitted March 1,
1834. Cough commenced at the end
of the Autumn of 1833. In two months
expectoration occurred, once or twice
only streaked with blood. In the Win-
ter, pain in the chest and emaciation
commenced. On his admission the
symptoms of phthisis were distinct.
Slight dulness on percussion at the
upper part of the right side of the
chest; feeble inspiration throughout
the upper two-thirds of that lung;
resonance of the voice much increased
at its upper and posterior part.
On the morning of the 30th of May,
he was awakened by severe pain in the
right side of the chest; when he turned
towards that side he felt something fall
from the inside of the chest towards
the ribs, with so much increase of pain
as to impede his breathing. The respi-
ration was extremely feeble throughout
the whole extent of the right side, but
the percussion was perfectly sonorous.
On the 4th of June he was able to
sit up. The right side of the chest was
found to be more prominent than the
left, it measured nearly two inches
more in semi-circumference. Percus-
sion gives a tympanitic sound over the
whole right lung. On the left side it
offers no changes. The respiration is
almost absolutely wanting on the right
side, a faint blowing inspiration is con-
fined to the top of the lung. The cough
and the voice both yield the peculiar
resonance termed amphoric, and a
metallic tinkling follows each effort of
coughing. On the left side the respi-
ration is expansive and natural through-
out, except near the clavicle, where it
is rough. Forty to fifty inspirations
per minute; pulse quick, 120.
From the 4th to the 9th the dyspnoea
increased, with decubitus on the left
side. On the morning of the 10th, he
died.
Dissection, nine hours after death.
" On perforating the right pleura above
the nipple, a stream of air escaped from
the opening with a whizzing sound,
and at the same time the intercostal
spaces evidently subsided. The right
pleura contained about a pint of
troubled serosity, mixed with flocculi
of lymph ; the costal pleura was not
reddened, but it was lined with a coating
of irregular false membrane, easily de-
tached from it. The lung was closely
applied to the mediastinum, and was
reduced to the thickness of an inch;
its pleura was covered with yellowish
lymph, about the thickness of a dollar,
which was partly disposed in a smooth
layer, and in other parts was of a cel-
lular aspect, like the false membrane
usually found in pericarditis. The
pleura was pale and wrinkled. The
summit of the lung was occupied by a
cavity the size of a small walnut; it
was nearly empty, and lined by a firm
cartilaginous membrane, with some
patches of a soft internal coating ad-
hering to it. An irregular perforation
communicating with the cavity of the
pleura, was seen near some strong ad-
hesions, which were limited to the vici-
nity of the tuberculous excavation. The
opening did not exceed a line in diame-
ter. The rest of the lung was of a
grayish colour, perfectly permeable to
the air, and contained numerous gray
and yellow granulations."
The left lung adhered at several points
near the summit. It had numerous
tubercles, and one cavity the size of a
large almond.
Guy's Hospital.
Analysis of the Organic Lesions
found in 100 Cases, in whicii
the Urine was Albuminous.*
When the mind is devoted to a par-
* Guy's Hosp. Reports, No. II.
1836] Guys Hospital Reports. 565
ticular investigation, it is difficult, per-
haps impossible, to avoid attaching an
overweening importance to the subject
which so fully occupies it. The mental
telescope, like the physical, enjoys only
a limited range, and if the object at
which it is pointed is distinctly seen, its
magnitude and clearness are observed
at the expense of others which become
no'longer visible. We know not whe-
ther this general truth has been con-
spicuous in the case of Dr. Bright. He
has investigated with much success the
conditions of the kidney, connected
with an albuminous condition of the
urine, but possibly he may have gone
a little too far in the consequences that
he has. drawn from his researches.
In the Guy's Hospital Reports, a
Well-written paperon albuminous urine,
which was noticed in our last number,
is followed by a tabular view of the
morbid appearances occurring in 100
cases, in which that state of the urine
existed. The tabular view is too long
for insertion, but we can afford space
for an analysis of its prominent fea-
tures.
A glance at the table is sufficient, as
Dr. Bright remarks, to convince us,
that many and important lesions are
the frequent accompaniments of the
granular condition of the kidney, to
which he has so particularly directed
attention. It is natural that Dr. Bright
should attribute to the kidney their oc-
currence?but it is difficult to deter-
mine the absolute correctness of this
idea without a careful and extended
consideration of all the facts of the
cases. For our own parts we cannot
help suspecting that Dr. Bright rather
over-rates the importance, if not the
frequency, of this renal disease. How-
ever that may be, we will present a
sketch of the lesions which were ac-
tually discovered in the 100 cases in
question.
A- Lesions in the Serous Membranes.
These display a considerable tendency
to inflammation. The pleura shews
?!" tfudeucy most distinctly.
" rhe disease has, in forty cases,
consisted of old adhesion; which,
though it might have been connected
No. L.
with the first attack of renal disease,
or might have taken place at some later
period, in connexion with that affection,
may probably only mark the liability of
the individual to be affected by atmos-
pheric changes, and may have been the
result of some casual inflammatory
attack. At all events, the twenty-six
cases in which the pleura was appa-
rently healthy, and in three of which
its freedom from disease is distinctly
stated, prove, that however general a
limited inflammatory action of the
pleura may have been, it forms no essen-
tial part of the disease. That the pleura
is, however, liable to inflammatory ac-
tion, in a large proportion of these
cases, may be inferred from the sixteen
instances of recent inflammation ; while
the serous effusion, which has occurred
in forty-one cases, has been connected
with that general loss of balance be-
tween the actions of the exhalents and
the absorbents which is obvious in every
part of the system.
The same tendency to disease which
is manifest in the pleura, shews itself,
though in a less degree, in other serous
membranes. In the pericardium, we
have found six instances of old ad-
hesion, eight of recent inflammation,
and twenty-three of serous accumu-
lation ; and in the peritoneum, ten in-
stances of old adhesion, twelve or thir-
teen of well-marked, recent, and often
most acute inflammatory action ; and
twenty-three of the effusion of clear
serum, in three of which a false mem-
brane had been formed by chronic
action : and again, looking to the arach-
noid, we find that membrane rendered
opaque, probably by a more or less
severe inflammatory action, in thirteen
cases ; while well-marked serous accu-
mulation had taken place beneath it in
twenty-nine cases, and had partially
distended the ventricles in six."
u. Lesions of the Heart.
This organ has shewn great liability
to alteration. In twenty-seven cases,
no disease could be detected, while in
six others it is probable that there was
none. The obvious structural changes
in the heart have consisted chiefly of
hypertrophy with or without valvular
P p
566 Periscope; pr, Circumspective Rbvirw. [Oct. I
disease : and what is most striking,
out of fifty-two cases of hypertrophy,
no valvular disease whatsoever could be
detected in thirty-four; but in eleven
of these thirty-four, more or less dis-
ease existed in the coats of the aorta;
still, however, leaving twenty-two with-
out any probable organic cause for the
marked hypertrophy generally affecting
the left ventricle. Dr. Bright suspects
that the altered state of the blood is the
cause of the hypertrophy, but this is
rather fanciful. When we recollect
that most of these cases occur in per-
sons in the lower class of life, who
abuse ardent liquors, a much more
probable explanation presents itself.
Hard work and alcoholic drinks are
probably the real stimulants of the
heart's action, and the efficient causes
of its hypertrophy. Independently of
renal disease altogether, ventricular hy-
pertrophy is a very common, perhaps
the most common lesion, discovered in
the lower class of people between the
ages of thirty-five and fifty. Our own
observation would lead us to think this,
but we are leaning upon that observa-
tion and not upon actual numerical
data. We would say that hypertrophy
of the heart is a much more common
disease than the granulated kidney, and
when they co-exist, the former is, cae-
teris paribus, likely, in many instances,
to have been the antecedent. But pro-
babilities of any sort are of little weight
in questions of this kind, where rigo-
rous observation should determine the
order of the phenomena.
The hypertrophy of the heart was
found to have kept pace with the ad-
vance of disease in the kidney. Six
cases are noted, in which the heart was
soft and flaccid, and four in which it
was unusually small; and in most of
these, though not in all, the disease of
the kidney had not proceeded to the
state of contraction and hardness.
c. Lesions of the Lungs.
" The principal diseases of the lungs
have been oedema and bronchitis, fre-
quently attended by an emphysematous
condition of certain portions. (Edema
has occurred in thirty-one cases ; and
it is very commonly the immediate cause
of dissolution, or of the increased dis-
tress towards the approaching termi-
nation of the chronic form of the dis-
ease. In six cases, recent, and in five
old, traces of pneumonia were found;
while the embarrassment to the circu-
lation, caused by these various diseases
of the heart and lungs, had occasion-
ally given rise to the effusion of blood
into the tissue of the lungs, in the form
which is now known by the term of
pulmonic apoplexy. The instances in
which phthisis, or any form of scrofu-
lous or tuberculous disease, has been
connected with the renal affection, have
been decidedly rare; so that in only
four cases has recent phthisis deve-
loped itself: and what is somewhat re-
markable, in more than double that
number the disease seems to have made
a certain inroad upon the upper lobes
of the lungs, and then to have sunk into
a state of quiescence, or entirely sub-
sided : from which we should perhaps
be inclined to infer, that so far from
these diseases being associated, the con-
dition of the body in this form of renal
disease is unfavourable to the existence
of phthisis, or that it is certainly not
peculiarly apt to occur in tuberculous
constitutions."
We think there can be little doubt
that the granular kidney is not the
product of the state of system that oc-
casions or that waits on phthisis. The
evidence is of a two-edged character.
In the first place, out of the numerous
cases of phthisis, we seldom see any
in which this renal alteration takes
place; in the second place, the latter
is seldom found to be attended with
phthisis. It is rather the product of
that constitutional condition occasioned
by intemperance, in which the heart is
more apt to be involved than the lungs
are.
d. Lesions of the Liver.
The following observations are im-
portant. They tend to correct a no-
tion which has been carried to an ex-
travagant length.
" With regard to the liver and the ab-
dominal viscera generally, as compared
with the heart and lungs, a very great
immunity from structural disease is to
be observed ; a fact the more remark-
1836] Guy's Hospital Reports. 567
able, as the habits of intemperance with
"which the renal disease is so frequently
connected are those which might be ex-
pected to act very directly on the liver
and digestive organs: indeed, to this
day, the impression is so strong, as to
the injurious effects of stimulants be-
ing manifested chiefly on the liver, that
the majority of practitioners no sooner
see the bloated countenance of anasarca
connected with the history of intem-
perance, than they proceed to consider
in what way the depraved action of the
liver is to be corrected, and its morbid
changes retarded. Looking to the tables
before us, a very different conclusion
forces itself upon our mind, as to the
condition of the liver in general ana-
sarca, and in that state of cachexia
Which often attends upon intemperate
habits. We here find, in thirty-one
cases, the liver distinctly stated to be
healthy; and in nine other cases, so
free from all suspicion of deranged ac-
tion, as to be pointed out as remarkable
specimens of the healthy organ; thus
making forty in the hundred free from
disease. In thirty-two cases, any de-
viation from the natural appearance was
exceedingly slight; and was, in a large
proportion of them, nothing more than
that mottled state which is derived
from the irregular distribution of blood
throughout the texture?a condition
very frequently observed, where the cir-
culation through the chest is obstructed.
The instances of confirmed diseased
structure did not amount to above eigh-
teen. There seemed to be no marked
connexion between the condition of the
kidney and of the liver; for nearly one
half of those cases which were stated
to be remarkably healthy were coupled
With the hard and probably most-ad-
vanced form of the disease, while the
other half occurred in cases apparently
less advanced; and the more severe
cases of hepatic derangement accom-
panied every variety of the disease in
the kidney. The only two instances
?f fatty degeneration in the liver were
m cases where the kidney was soft,
smooth, and white; but in another,
where the liver was somewhat fatty,
the kidney was hard, rough, and lobu-
lated." '
e. Lesions of the other Abdominal
Viscera.
The stomach seems to have suffered
in some measure from the abuse of sti-
mulants. In eighteen cases the mu-
cous membrane is recorded to have
suffered from irritation. In many more,
perhaps its condition was not accu-
rately ascertained.
In about nineteen cases, the small
intestines have been irritated in some
portions of their course?in a few of
these, ulceration has taken place ; and
in seven cases the colon or caecum has
been diseased; but several of these have
occurred in conjunction with tubercles
in the lungs, and have therefore been
scarcely ascribable to the peculiar cir-
cumstances of this disease.
f. Lesions of the Brain.
These have chiefly consisted of that
unequal distribution of blood which is
apt to produce a mottled appearance
when the medullary substance is exposed
in slices, and which is frequently at-
tendant on convulsive or apoplectic sei-
zures. In some cases, the brain has
been exsanguine ; and in a few, the re-
sults of such lesions as the rupture of
vessels may induce, have been observed.
G. Immediate Causes of Death.
In seventy cases Dr. B. has been able
to determine the immediate cause of
death. No less than thirty out of these
seventy have died of well-marked symp-
toms of cerebral derangements, noted
under the titles of ' apoplexy/ ' coma,'
' convulsion,' and ' epilepsy.' Eight
others have died suddenly. In eight
cases, the obstructed condition of the
lungs has been the immediate cause of
death; and in three, the effusion into
the chest has hastened the dissolution.
Next to head affections, the most pre-
valent diseases have been inflammatory
attacks in the serous membranes ;?
amongst which are five well-marked
cases of peritonitis, three of pericardi-
tis, one or two of pleuritis. Diarrhoea
and other exhausting diseases have car-
ried off several.
h. Age at rvhich Death occurs.
The Table tends in a material degree
lJ p 2
568 Pkriscopk; or, Circumspective Rkvikvv. [Oct. 1
to illustrate this curious and important
point.
" In seventy-four cases, the age has
been recorded ; and of these, four only
have survived beyond their sixtieth year;
thirteen have passed their fiftieth year ;
but few of them have lived to fifty-five :
twenty-one have died between forty
and fifty ; sixteen have passed thirty
years ; and nineteen have died before
they had arrived at their thirtieth year :
and if we take those who have died in
their forty-fifth year and belowthat age,
we find that the large proportion of
fifty out of seventy-four have sunk be-
fore the meridian of life. The youngest,
whose age is given, is only eight; and
there is one advanced to seventy-three :
shewing, therefore, that neither youth
nor age is exempt from this disease,
but that it has cut off the greater part of
its victims before the middle period has
been attained."

				

